# Integrative Analysis of Low- and High-Resolution eQTL

**DOI:** 10.1371/journal.pone.0013920

**Published:** 2010-11-10

**Authors:** Salvatore Loguercio, Rupert W. Overall, Jacob J. Michaelson, Tim Wiltshire, Mathew T. Pletcher, Brooke H. Miller, John R. Walker, Gerd Kempermann, Andrew I. Su, Andreas Beyer

**Affiliations:** 1 Biotechnology Center, Technische Universität Dresden, Dresden, Germany; 2 Center for Regenerative Therapies Dresden (CRTD), Technische Universität Dresden, Dresden, Germany; 3 Division of Pharmacotherapy and Experimental Therapeutics, University of North Carolina School of Pharmacy, Chapel Hill, North Carolina, United States of America; 4 Compound Safety Prediction, Pfizer Global Research and Development, Groton, Connecticut, United States of America; 5 Department of Neuroscience, The Scripps Research Institute, Scripps Florida, Jupiter, Florida, United States of America; 6 Genomics Institute of the Novartis Research Foundation, San Diego, California, United States of America; Cairo University, Egypt

## Abstract

The study of expression quantitative trait loci (eQTL) is a powerful way of detecting transcriptional regulators at a genomic scale and for elucidating how natural genetic variation impacts gene expression. Power and genetic resolution are heavily affected by the study population: whereas recombinant inbred (RI) strains yield greater statistical power with low genetic resolution, using diverse inbred or outbred strains improves genetic resolution at the cost of lower power. In order to overcome the limitations of both individual approaches, we combine data from RI strains with genetically more diverse strains and analyze hippocampus eQTL data obtained from mouse RI strains (BXD) and from a panel of diverse inbred strains (Mouse Diversity Panel, MDP). We perform a systematic analysis of the consistency of eQTL independently obtained from these two populations and demonstrate that a significant fraction of eQTL can be replicated. Based on existing knowledge from pathway databases we assess different approaches for using the high-resolution MDP data for fine mapping BXD eQTL. Finally, we apply this framework to an eQTL hotspot on chromosome 1 (*Qrr1*), which has been implicated in a range of neurological traits. Here we present the first systematic examination of the consistency between eQTL obtained independently from the BXD and MDP populations. Our analysis of fine-mapping approaches is based on ‘real life’ data as opposed to simulated data and it allows us to propose a strategy for using MDP data to fine map BXD eQTL. Application of this framework to *Qrr1* reveals that this eQTL hotspot is not caused by just one (or few) ‘master regulators’, but actually by a set of polymorphic genes specific to the central nervous system.

## Introduction

Understanding how genetic variability affects molecular phenotypes is important for revealing the molecular mechanisms underlying physiological phenotypes such as complex diseases [1]. Genome-wide measurements of RNA expression levels as molecular phenotypes have been correlated to genetic variation in a variety of biological contexts [2]–[6]. Such data are used for the identification of expression quantitative trait loci (eQTL), i.e. genomic regions whose genotype is correlated with the RNA expression in a panel of genetically diverse individuals [7]. The underlying assumption is that such eQTL regions contain direct or indirect regulators of the target gene in question.

The considerable advances in eQTL studies notwithstanding, there are still open questions about the biology and applications of eQTL mapping [8]. One persistent challenge in the field is the selection of loci and genes that should receive further biological investigation. For instance, eQTL are often only poorly reproducible across studies [9]. The difficulty to reproduce eQTL (and other genetic associations) is partly explained by differences between the study populations: different populations have different genetic backgrounds, which may considerably alter the phenotypes associated with a given allele. Secondly, the genome-wide measurement of RNA levels is noisy, which may result in many spurious eQTL. Finally, because of the large number of statistical hypotheses tested in eQTL studies (all genes versus all markers) such studies notoriously suffer from high false-positive rates unless stringent controls for multiple testing are applied.

In order to overcome these problems, recent studies have combined different model populations in their studies, thereby enhancing genetic resolution, power of the study and reproducibility of the findings [10], [11]. The reproducibility of eQTL in two populations of closely related mice, BXD recombinant inbreds (RI) [12] and an F2 cross between the same parental strains has been reported to be high [13]. However, it is not clear to which extend eQTL will replicate in more diverse panels within the same species. Recently, Gatti et al. [10] compared mouse liver eQTL from BXD RI lines and inbred strains of the Mouse Diversity Panel (MDP). Consistent with other reports, they found that *cis*-eQTL replicate with greater frequency than *trans*-eQTL, although, a systematic, quantitative assessment of the extent of consistency between the two datasets is lacking.

Another major problem of current eQTL studies is the correct identification of the causal genes. Due to linkage disequilibrium, several genes can be near each marker making it difficult to identify which of the genes are the causal factors responsible for the observed expression phenotype. While linkage studies in F2 or recombinant inbred (RI) lines are thought to have reasonable statistical power, the limited number of recombination events in such crosses produces large QTL intervals that make the selection of candidate genes difficult. One approach that has been used to narrow individual QTL intervals is *in silico* haplotype mapping in laboratory inbred strains [14], [15]. Other approaches utilize additional, independent information such as protein interaction data in order to identify causal genes [16]–[18]. For a single phenotype, several methods have been described to combine QTL data from different crosses [19], [20]. However, due to the high cost of replicating a large eQTL study and the computational issues of combining data for thousands of transcripts, these methods are difficult to apply to most eQTL studies. Using genetically more diverse populations has the advantage that a higher density of informative genetic markers can be achieved, which potentially increases the mapping resolution. Unfortunately, those studies often suffer from poor statistical power [21].

Thus, it has been suggested that eQTL obtained from populations with greater genetic diversity, such as the MDP, should be combined with RI population-based data [10], [11], [22]. The underlying idea is to use the RI population-based data for identifying potentially causal loci and then use the high-resolution data for ‘fine mapping’ the causal gene. While there is evidence that this data integration helps in identifying causal genes, a systematic analysis of its potential has not been performed. It remains to be shown that high- and low-density eQTL data are consistent and that combining them aids the robust identification of truly causal genes.

In order to address these open questions, we investigated the reproducibility and fine mapping of eQTL based on BXD RI lines [23] and inbred strains of the MDP [21]. We measured hippocampus gene expression in 28 strains from the MDP and compared and integrated those data with corresponding eQTL data from the BXD panel [24]. We systematically show consistency between the two datasets for a wide range of eQTL thresholds and discuss various strategies to integrate high- and low-resolution eQTL data for fine mapping purposes. We assessed those data integration schemes for fine mapping by quantifying the ability of each method to reproduce known functional associations between genes. Finally we used the MDP-based data to improve the genetic dissection of a brain-specific eQTL hotspot on distal Chromosome 1 [25].

The actual density of markers is not the limiting factor for the genotype resolution in the two populations. While, BXD and MDP have been genotyped at virtually the same markers, the smaller number of recombination events in the BXD population creates larger chromosomal regions that are inherited from the same parent. Consequently, few markers are actually informative; that is, several adjacent markers carry the same information about the origin of a particular genomic region. In the remainder of this text we use the terms ‘MDP markers’ and ‘BXD markers’ to denote those markers that are informative in the respective populations. Note that the majority of BXD markers are part of the MDP markers (3055 out of 3791, 80%).

## Results and Discussion

### Consistency between MDP and BXD eQTL datasets

An overview of eQTL datasets considered in this study is provided in Table 1. In order to investigate the reproducibility of eQTL between independent mouse populations, we compared the number of eQTL common between the MDP and BXD datasets to the number of common eQTL obtained with randomized data.

**Table 1:** Overview of eQTL datasets used.

Dataset	BXD eQTL dataset	MDP eQTL dataset
Experimental source	Overall et al. [24]	This study a
Genetic reference panel	BXD	Mouse Diversity Panel (MDP)
Number of strains	71	28
Number of microarrays	201	28
Number of markers	3,791	101,104
Number of probe sets	45,101	44,922
eQTL detection method	QTL Reaper [13]	Haplotype Association Mapping (HAM) [51]

a Genomics Institute of the Novartis Research Foundation (San Diego, CA, USA).

**Figure 1:**
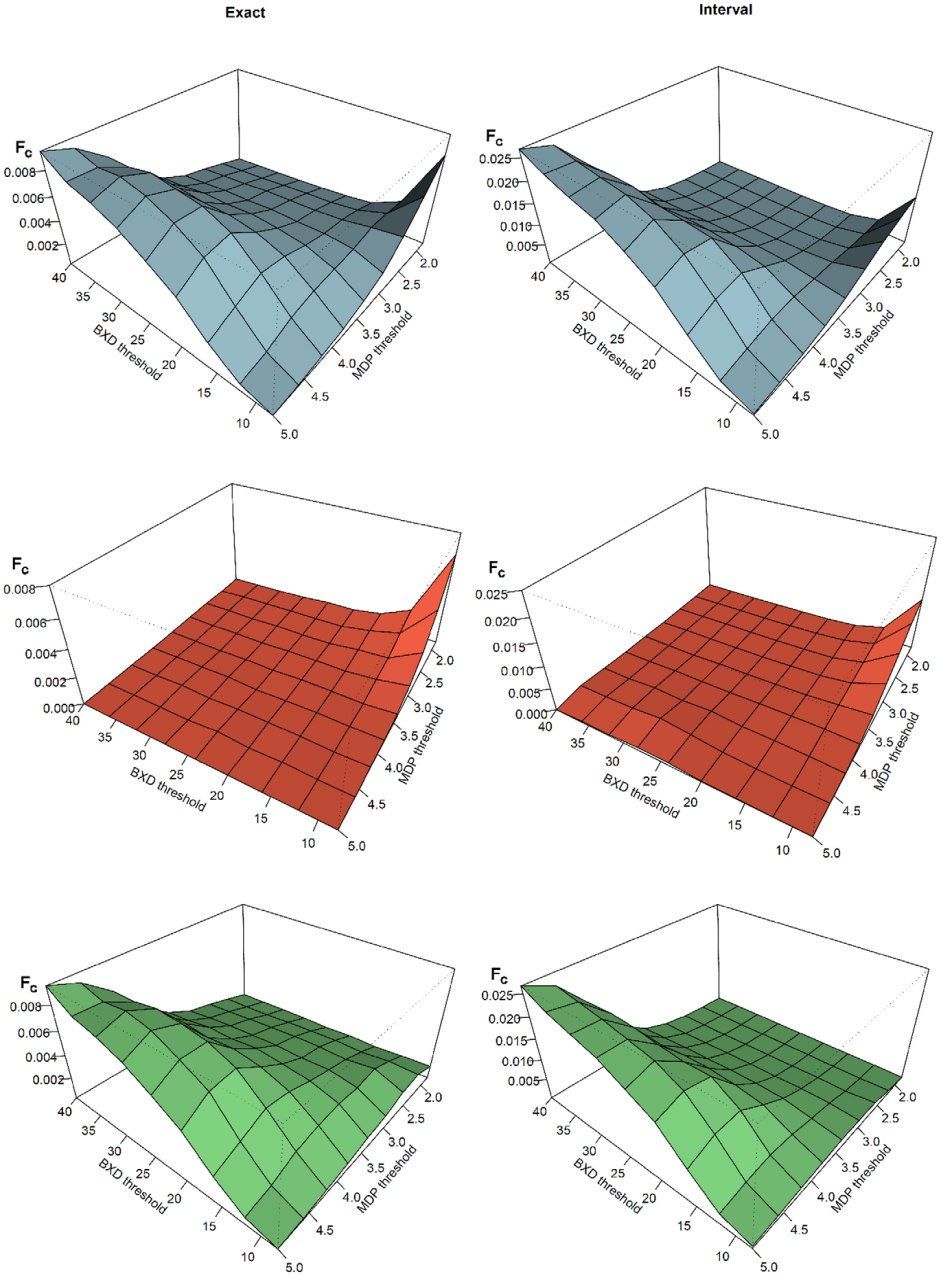
Consistency between MDP and BXD eQTL datasets. Average fraction of common eQTL signals, *F_C_* = *mean(N_HITS_/(N_MDP_+N_BXD_))*, as a function of BXD and MDP signal detection thresholds. The exact comparison (left) compares signals at identical markers in the two populations, whereas the interval comparison (right) summarizes the MDP values in proximity of BXD markers. **Top panels**: actual measurements. **Middle panels**: shuffled comparisons (10 randomizations). **Bottom panels**: difference between real and shuffled data for exact and interval comparison.

All 25,173 probesets with unique genomic locations shared between the BXD and MDP datasets were included in this comparison. We tested two strategies comparing the eQTL scores: the exact comparison directly compares the scores of common markers, whereas the interval mapping integrates the information from several (high-resolution) MDP markers near (low resolution) BXD markers (Figure 1, Table 2). At very high quantile cutoffs (99.99% or higher) between 1% and 2% (65–165) of the BXD eQTL could be replicated in the MDP population. Even though this is only a small fraction of all eQTL, the overlap is much larger than expected by chance (genome-wide comparison; Fisher's exact test p-value at 99.99% quantile cutoff: <1×10^−4^ for the exact comparison, 5×10^−4^ for the interval comparison).

**Table 2:** Consistency of eQTL datasets.

Quantile cut-off	0.99	0.999	0.9999	0.99995
Number of selected eQTL (BXD)	956,272	95,468	9,545	4776
Number of selected eQTL (MDP)	25,357,555 (953,407)^a^	2,523,474 (95,121)	252,525 (9,605)	126,068 (4,801)
Number of common eQTL (*N_C_*)	5,897 (8,295)	416 (572)	106 (165)	65 (101)
Fraction of *N_C_* with P-value < = 0.05	49.0% (34.5%)	100%	100%	100%
*N_C_/N_BXD_* b	0.006 (0.0087)	0.0044 (0.006)	0.011 (0.017)	0.014 (0.021)

a Values in parentheses refer to interval comparison.

b *N_C_*, number of common eQTL; *N_BXD_*, number of selected eQTL in BXD dataset.

Next, we extended the consistency analysis over a continuous range of thresholds. Figure 1 shows the fraction of common eQTL as a function of both the BXD and MDP eQTL scores. In order to show the significance of the overlap we compared the fractions of overlapping eQTL resulting after randomizing the data. We binned BXD and MDP eQTL scores, thereby establishing a two-dimensional grid of the two scoring schemes. Each bin in this grid contains eQTL with scores comparable within BXD and within MDP data. For the randomization we shuffled the BXD and MDP scores within each bin by re-assigning them to different target genes. We then compared the number of ‘common’ eQTL in each randomized bin to the observed number. Figure 1 clearly shows that the overlap expected by chance is substantially smaller than the observed overlap for a wide range of thresholds.

The fraction of common signals in the actual measurements is always higher than the shuffled one, both in the exact and interval comparison schemes. In the interval scheme, the average number of common peaks is approximately three times higher, but the signal-to-noise ratio – i.e. the difference between the fraction of correct and shuffled comparisons – is smaller over the entire range of thresholds. Hence, the interval mapping yields a higher recovery at the cost of lower specificity (more false positives).

**Figure 2:**
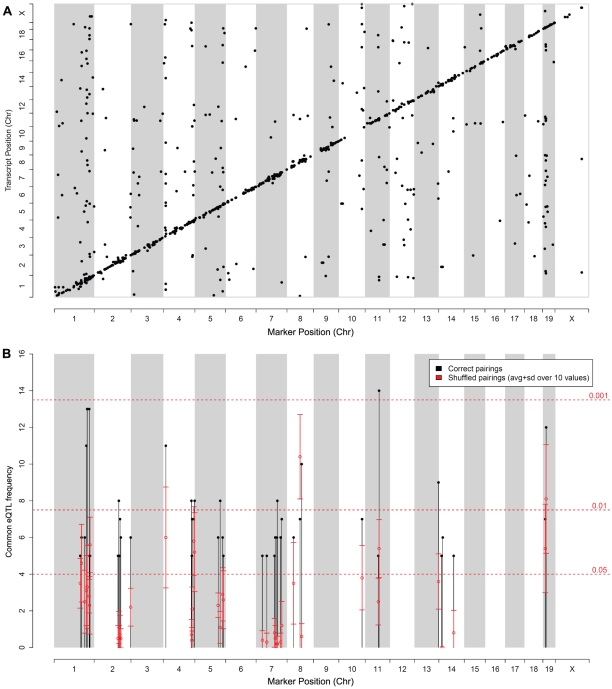
Co-localizing common eQTL between MDP and BXD datasets. The top 0.5% of all eQTL of the two datasets were compared using the ‘exact’ comparison and common eQTL are shown. **A**: matrix of common eQTL. X-axis shows marker positions and y-axis shows target gene positions. Each dot corresponds to an eQTL common between the two studies. **B**: Number of common eQTL across the genome within 1320 equally sized bins of 2 Mb. Black bars report the number of common eQTL in each bin, whereas the red error bars represent the mean number and standard deviation of hits in each bin obtained after shuffling the probesets (10 randomizations). Horizontal red lines represent global p-values obtained after shuffling bin positions. Results are shown for bins with at least 5 common eQTL.

Figure 2 shows the genomic distribution of common eQTL after the exact comparison at the 99.5% quantile cutoff (1,071 matches with comparison p-values below 0.05). The matrix of common eQTL in Figure 2a shows that local (*cis*-) eQTL are much more reproducible than distant (*trans*-)eQTL. The majority of common eQTL localizes at the diagonal or in its immediate vicinity. Local eQTL linking a target gene to its own locus are assumed to be due to polymorphisms acting in *cis*, e.g. in the promoter of the gene (hence the name *cis*-eQTL). Distant eQTL, on the other hand, are due to polymorphisms in genes other than the target gene. Such *trans*-eQTL exerts its effect through variable activity of a regulator controlling the target. The overrepresentation of *cis*-eQTL in Figure 2 is consistent with the fact that, in general, *cis-*eQTL tend to produce stronger statistical associations than *trans-*eQTL [26] and are easier to reproduce [10], [13].

For each marker we computed the number of eQTL common between BXD and MDP (‘common eQTL frequency’, CEF). Plotting the CEF across the genome (Figure 2b) indicated a non-random distribution with some hotspots along the genome (black bars). Among these eQTL-hotspots is a well-studied hotspot on chromosome 1 [25]. To assess the significance of these hotspots, we randomized the data in two different ways: first by shuffling marker positions and second by randomizing probeset assignments. The first randomization shuffled the assignment of eQTL to genomic positions (i.e. markers), yielding global p-values for the observed frequencies (dotted red lines in Fig. 2b). In the second scheme, we randomized the probe set assignment of the eQTL at each locus, i.e. eQTL from MDP were compared against random probesets from BXD. This second approach accounts for the fact that some loci have large numbers of eQTL, which may yield large CEF simply by chance. However, the shuffled values generally yield few common eQTL (the real values are higher than the shuffled values in 39 out of the 44 peaks shown in Figure 2b). In some cases the number of observed overlapping eQTL is much higher than expected by chance (e.g. on chromosomes 1, 4, 8, 11 and 19). However, when less strict eQTL thresholds are used, we could observe a similar genomic distribution of CEF for correct and shuffled comparisons (data not shown). This suggests that several *trans*-bands observed at high-confidence thresholds are common to MDP and BXD and are of biological significance. This is an important finding, as eQTL-hotspots are often spuriously identified due to batch-effects or population sub-structure [27]–[29]. Since the hotspots in Figure 2 were reproduced in two different populations they are much less likely to be artifactual. Taken together, these observations suggest that the MDP and BXD eQTL are consistent, that high-scoring eQTL can be reproduced and that several eQTL hotspots are conserved across the two mouse panels.

### Integration of MDP and BXD eQTL data for fine mapping

Given the high statistical significance of the overlap between the two populations, we sought to use the high-resolution MDP data for fine mapping causal genes in loci that are significant according to the BXD panel. Given a locus with several candidate genes, we wanted to use the MDP data to refine the identification of the causal gene. Here we considered a situation where a marker is strongly linked to some target gene based on the BXD data. All genes between the two flanking markers from the BXD dataset are putative transcriptional regulators. The higher density of informative markers in the MDP population may help to select the most likely causal gene.

**Figure 3:**
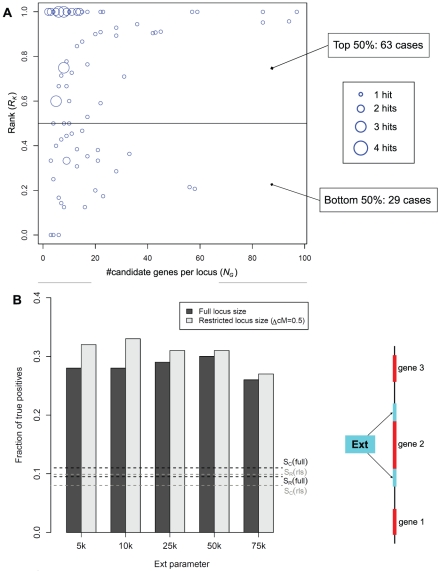
Integration of MDP and BXD eQTL data for fine mapping. **A**: Distribution of the ranking results for 105 selected eQTL intervals with strong association signals in both the BXD and MDP datasets. The x-axis shows the number of candidate genes at each locus and the y-axis shows the rank (scaled between 0 and 1) of genes that are in common pathways with the target genes. Thus, each eQTL interval is represented as a point of coordinates (*R_K_, N_G_*) where *R_K_* is the rank of the (presumed) causal gene from the KEGG pathway and *N_G_* is the number of genes within the interval. Circle sizes are proportional to the number of eQTL. Pathway co-members rank significantly above random when using the MDP data as additional information for scoring candidate genes. **B**: Assessment of MDP-based candidate gene selection. For each of the 105 selected loci a selected candidate gene was considered as a true positive if it is part of a common pathway with the target gene. The figure shows the fraction of loci with correct selections. Bars show results when ranking genes based on their MDP scores for different values of the *ext* parameter (see schema on the right), using full locus and restricted locus sizes (i.e. loci are restricted to 0.5 cM around the BXD marker). Horizontal lines show true positive rates for random selections (S_R_) and selecting the closest gene (S_C_). Black lines: ‘closest gene’ success rate (S_C_) and random success rate (S_R_) for full locus sizes. Grey lines: S_C_ and S_R_ for restricted locus sizes (rls).

In order to assess the performance of the MDP-based fine mapping, we decided to restrict the analysis to eQTL with very strong association signals in both the BXD and MDP datasets (see Materials and Methods). Application of our selection criteria yielded a list of 105 suitable eQTL intervals, which we subsequently used for assessing the MDP-based fine mapping. We scored the performance of our fine mapping based on whether the selected gene from the locus is annotated as a member of the same pathway as the target gene. Using the pathway annotation from the Kyoto Encyclopedia of Genes and Genomes (KEGG) we quantified the number of suggested causal genes being annotated for the same pathway as the target gene of the respective eQTL (see Materials and Methods for details). This assessment is based on the assumption that the source gene (i.e. the QT gene) and the target gene are likely to be involved in the same biological process. Hence, methods yielding a higher enrichment for genes being members of the same pathway gain higher credibility. According to this criterion causal genes should rank high (be on top of the list) when sorted based on the MDP eQTL. Figure 3a shows that MDP-based fine mapping ranks functionally related genes higher in the list than expected by chance (top 50%: 63 cases; bottom 50%: 29 cases; not ranked: 13 cases). Figure 3b shows that genes from the same pathway are significantly more often ranked highest when using the MDP-based mapping as opposed to picking the gene closest to the BXD marker or a random gene from the locus. When using the MDP data the pathway members rank highest in 33% of the cases, as opposed to 8% when selecting the gene that is closest to the BXD marker (S_c_) and 10% when choosing a random gene from the locus (S_R_). These numbers include several *cis*-eQTL, hence, in those cases the causal and target gene are identical. However, since our fine-mapping algorithm did not consider location information about target genes, there is no particular advantage (or bias) for resolving causal genes in *cis*-eQTL regions except for the fact that *cis*-eQTL in general tend to be stronger. In order to demonstrate that the enrichment of true causal genes is independent of *cis*-eQTL we hence repeated the analysis excluding all *cis*-eQTL (Figure S1). Due to the weaker eQTL signals we did in fact observe a decrease of the overall performance when exclusively looking at *trans*-eQTL. Yet, using the MDP data for fine mapping still significantly improved the identification of causal genes. Thus, even though the MDP data do not always correctly predict the most likely causal gene, the data substantially improve the gene selection.

Generally, we rank the genes within each locus based on MDP markers positioned inside the transcribed region of the genes. However, in cases when no MDP marker is located in the transcribed region we assign scores based on markers in the proximity of the genes. If there is no marker in the proximity of a gene that gene remains unscored and it is not considered as a causal gene (see Materials and Methods for details). The definition of this ‘search region’ is a critical parameter that affects the prioritization of genes within a locus, as demonstrated in Figure 3b. When the search region is too small, many genes remain unscored and are thus not ranked, yielding a high false negative rate. If, on the other hand, the search region is too large, markers are considered that are too far away from the respective gene and thus unlikely to correctly map the genotype of the gene.

In addition, we observed a correlation between the strength of MDP eQTL scores and their ability to correctly map causal genes. Figure S2 indicates that the consideration of lower-scoring eQTL yields smaller fractions of pathway members that are ranked first. The fact that the performance deteriorates at lower scores underlines once more that the consistency of high MDP scores with pathway membership is not by chance. Thus, high-scoring MDP eQTL can be used for improving the detection of causal genes; however, weaker MDP scores should be used with caution.

### Defining the locus

If no additional information is available, one has to assume that the genetic variation causing the phenotypic change (i.e. expression change) may be located anywhere between the two markers neighboring the linked marker [30]. However, if the genetic distance between the linked marker and the flanking markers is large, it is less likely that the causal genetic variation is far away from the linked marker (i.e. close to the flanking markers). Thus, in order to further improve the causal gene identification it may be useful to only consider genes at a limited genetic distance away from the linked marker.

For testing this hypothesis we limited the set of genes considered at a given locus by a maximal genetic distance (see Materials and Methods for details). We defined eQTL intervals via genetic distances expressed in centimorgans (cM) in order to restrict interval sizes and the number of genes associated with each selected marker. Then we re-ran the fine mapping analysis as described before at different ΔcM (0.1, 0.3, 0.5, 0.7, 1, 1.5, >1.5), while simultaneously varying the search region around each gene. Figure 3b and Figure S3 show that restricting the locus size yields consistently better results as opposed to considering all genes between the flanking markers. Optimal performance was obtained with 0.5 cM.

### Using MDP eQTL to dissect an eQTL hotspot on distal Chromosome 1

Several eQTL studies with intercross or RI strains have observed that many distant eQTL map to the same location on a chromosome, giving rise to what is known as eQTL hotspots or *trans*-bands [24], [31], [32]. In general, the presence of eQTL hotspots suggests either the presence of a master regulator gene which controls the expression of groups of genes together or the presence of several tightly linked genes within the hotspot, each of which regulates the expression of a subset of genes mapping to this locus [31], [33], [34]. Here we illustrate how the MDP eQTL dataset can be used to improve the resolution at a previously studied hotspot on distal chromosome 1. This analysis consists of two major parts: first we performed the MDP-based fine mapping as outlined above on a small set of eQTL with very high BXD and MDP eQTL. This analysis identified 14 local eQTL that could be reproduced in the MDP population and it identified high-confidence causal genes. In the second part we relaxed the criteria for inclusion of MDP eQTL in order to identify functional enrichment of genes mapping to sub-regions of *Qrr1*.

The distal part of mouse chromosome 1, (172.5–177.5 Mb) harbors 16 BXD markers and 122 known genes (Mouse Genome NCBI m37). It is also known as *Qrr1*, “QTL-rich region on chromosome 1”, as it contains a large number of previously identified QTLs for behavioral traits, including open field activity [35], fear conditioning [36], rearing behavior [37], and other measures of emotionality [38], [39]. In addition to behavioral phenotypes, a number of other metabolic, physiological and immunological traits have been repeatedly mapped to this region [25]. Of particular interest is the association between *Qrr1* and the response to a wide range of neuroactive drugs [40]–[43].

### Fine mapping Qrr1

*Qrr1* can be broadly divided into a gene-rich proximal part (*Qrr1p*, 172.5–174.5 Mb) and a relatively gene-sparse distal part (*Qrr1d*, 174.5–177.5 Mb). Recently, Mozhui et al. [25] revealed multiple distinct loci in *Qrr1* that regulate gene expression specifically in the central nervous system (CNS). In particular, they focused on *Qrr1d* and proposed *Fmn2*, *Rgs7* and a cluster of tRNAs as strong candidate regulators of this distal part of *Qrr1*. However, due to the limited resolution, they could not subdivide transcripts that map to *Qrr1p* into smaller functional modules.

Based on the fact that *Qrr1* has 254 informative markers in the MDP dataset we expected that incorporating the MDP-based eQTL in the analysis might further resolve the structure of this region. We first selected targets with strong BXD eQTL in *Qrr1*, and then examined the same set of target transcripts in MDP to better refine their eQTL positions. In BXD, there are 307 genes with maximal LOD scores on markers located in *Qrr1*. In order to obtain a set of high-confidence *Qrr1* target genes we filtered 205 genes having eQTL above the 99.9% quantile (of the entire eQTL matrix) for the subsequent analysis. In BXD, this cutoff corresponds to transcripts with a minimum LOD score of 4 (genome-wide p-value of approximately 0.01). In order to apply the fine-mapping procedure described above we further filtered this list for targets with MDP scores above the 99.9% quantile (37 eQTL).

Of the 182 BXD eQTL, 22 have a local eQTL in *Qrr1* and 11 of these local eQTL could be replicated in MDP (Table S1). Importantly, in 4 of those cases the maximum MDP score was at the target gene itself and in the remaining 7 cases it was very close (<500 kb) to the target gene. This suggests that most or even all of the causal mutations in Qrr1 act in *cis*.

The MDP-based fine-mapping further identified 26 *trans*-eQTL mapping to 14 putative regulators (or QT genes) in *Qrr1*. Only 4 of these regulators do not have a *cis*-eQTL themselves (*Apoa2*, *Fcgr2b*, *Olfr220*, *Hsd17b7*). However, upon closer inspection we noticed that all four of the candidate QT genes had another gene with a *cis*-eQTL nearby (<100 kb, see Table S1 for details). Hence, if these other genes were causing the respective eQTL, all causal genes in *Qrr1* would be affected in *cis*.

**Figure 4:**
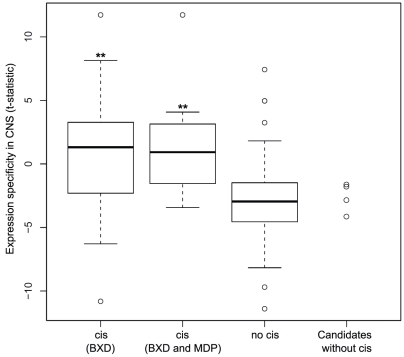
CNS expression specificity of candidate regulators in *Qrr1*. CNS specificity of expression is expressed as t-test statistics comparing expression in CNS tissues against all tissues tested. Box plots show distributions of t-scores for genes mapping to Qrr1. ***cis*** **(BXD)**: subset of genes with a *cis*-eQTL in BXD; ***cis*** **(BXD+MDP)**: genes with a *cis-*eQTL in both BXD and MDP; **no** ***cis***: genes localized in *Qrr1* but without a *cis-*eQTL; **Candidates without** ***cis***: candidate causal genes (distant eQTL only) without a *cis*-eQTL.

In order to further corroborate these candidate regulators, we integrated independent gene expression information. Mozhui *et al.* [25] had already shown that the *Qrr1* is specific for the CNS. Hence, we suspected that the genes causing the transcript variation of *Qrr1* target genes should be specifically expressed in the CNS. In order to confirm this hypothesis we checked whether genes with *cis*-eQTL are more specifically expressed in the CNS than others, which indeed is the case (Figure 4). However, the 4 candidate regulators without *cis*-eQTL only have poor CNS specificity of their expression (Figure 4) rendering them even less likely to be truly causal. Whatever the true QT genes are in these cases, we can conclude that most eQTL in *Qrr1* are ultimately caused by *cis* effects, since virtually all high-significance eQTL could be associated with a gene having a *cis*-eQTL.

**Figure 5:**
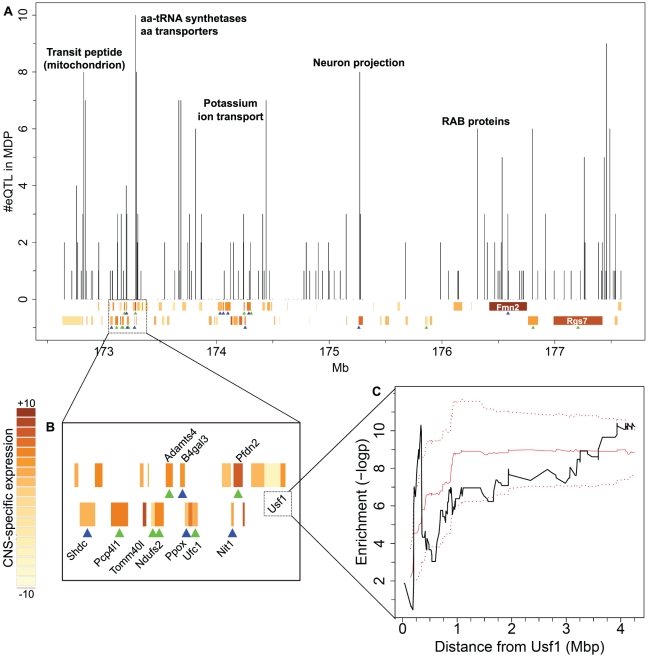
Using MDP eQTL to dissect an eQTL hotspot on distal Chromosome 1. **A**: Overview of candidate regulators number of eQTL mapping to *Qrr1*. Vertical bars show the number of target genes per marker in MDP (for every gene, the strongest eQTL in MDP within *Qrr1* is selected). Labels at the bars illustrate enriched functional categories. Genes encoded in *Qrr1* are shown at the bottom (top: forward strand, bottom: reverse strand). The color gradient reflects CNS expression specificity (t-statistic). Green triangles indicate *cis*-eQTL in the BXD dataset, whereas blue triangles represent a *cis-*eQTL detected in both BXD and MDP datasets. **B**: Detail of *Qrr1p* around 173 Mb. Labels of selected genes are shown. **C**: Enrichment of *Usf1* targets along *Qrr1*. Black line: enrichment (-logp) as a function of physical distance from the transcription factor, i.e. the length of an exploratory interval centered on *Usf1*. Red lines show results for randomized *Usf1* targets (Solid red line: average across 100 randomizations; dotted red lines: average ± standard deviation).

For the remaining analysis we relaxed the MDP eQTL threshold in order to increase the coverage, i.e. we considered all eQTL with an MDP eQTL above the 95% quantile (182 eQTL, the BXD cutoff was not changed). The larger number of eQTL considered allows us to assess whether eQTL target genes are enriched for known target genes or for certain functional categories. Figure 5 shows the location of MDP markers in *Qrr1* along with the respective number of target genes per marker. This analysis revealed an intricate sub-structure of *Qrr1*: there are distinct sub-regions having significantly larger numbers of target genes than other regions of *Qrr1*. Importantly, Figure 5 reveals that *Qrr1* consists of several eQTL hotspots. The fact that *Qrr1* harbors several causal genes is also supported by the robust detection of 11 local QTL in both mouse populations.

### *Usf1* regulates many *Qrr1* target genes

*Qrr1* encodes two transcription factors, *Atf6* and *Usf1*, while only the latter has a significant number of eQTL mapping to it. Hence, we set out to validate whether *Usf1* might be truly causal for these eQTL. *Usf1* is an essential transcription factor encoded in *Qrr1p* (approximately 173.35 Mb, Figure 5). Plaisier et al. [44] had measured the genome-wide response to over-expressing *Usf1*. This data provides independent experimental validation of *Usf1* target genes. Overall, there are 50 *Qrr1* target genes confirmed with the BXD eQTL data (Fisher's exact test p-value = 8×10^−6^). Assuming that *Usf1* is actually causal for several eQTL, we hypothesized that its target genes should be enriched among eQTL mapping close to *Usf1*. Hence, we used this notion to assess the resolution obtainable with the MDP data. In Figure 5c, the enrichment of known *Usf1* target genes (Fisher's exact test) is reported as a function of the physical distance from the transcription factor, i.e. the length of an exploratory interval centered on *Usf1*. A significant enrichment occurs within 300 kb from the gene; this result is confirmed by repeating the analysis at different thresholds in the BXD and MDP datasets, as well as different cutoffs for defining targets from the over-expression experiment (Figure S4). In order to demonstrate the specificity of this finding, we repeated the analysis with a non-candidate transcription factor in *Qrr1* (*Atf6*) with an associated dataset of differentially expressed genes after over-expressing *Atf6* [46]. Only 13 transcripts could be mapped in this case (Fisher's exact test p-value = 0.02), without enrichment in target genes at the *Atf6* locus (Figure S4). These results confirm that a mapping resolution of a few hundred kilobases is achievable with the MDP data. Many of the *Usf1* target genes mapping to this locus may be indirectly affected by the *Usf1* allele, i.e. they are not directly regulated via *Usf1* binding in their enhancer regions [44]. To address this question and to further validate our conclusions we utilized *Usf1* DNA binding data measured with ChIP-Chip in human hepatocytes [45]. Binding of *Usf1* was detected for 22 orthologous genes mapping to *Qrr1*, 13 of which mapped to the *Usf1* locus (within 300 kb). This is a highly significant enrichment (Fisher's exact test p-value <0.004), demonstrating that many of the genes affected by the *Usf1* polymorphism might in fact be direct *Usf1* targets.

### Target genes mapping to *Qrr1* sub-regions are functionally distinct

Provided that several causal genes contribute to the *trans*-eQTL observed in *Qrr1* we reasoned that different parts of this region should target functionally different groups of genes, i.e. the target genes mapping to different loci in *Qrr1* should be enriched for different functions. To test this hypothesis we selected functional categories that are significantly enriched among *Qrr1* target genes compared to the rest of the genome and checked whether those categories are enriched among target genes mapping to specific sub-regions of *Qrr1*. A complete list of all functional categories considered can be found in Table S2. In order to resolve marker-specific enrichment of functional classes, we computed the average MDP eQTL score at each marker for all genes of a specific class. This analysis was repeated in two different ways: first with all genes from a functional class with a significant BXD eQTL and secondly for the subset having a significant MDP eQTL as well. However, both approaches yielded comparable results (Figure S5). This analysis showed that several sub-regions of *Qrr1* are enriched for targets from specific functional classes, some of which are indicated in Figure 5. See Figure S5 for a complete overview.

Several mitochondrial genes have eQTL mapping between 172.7 and 173.3 Mb in *Qrr1*; ten of these genes possess a mitochondrial transit peptide, which is required for transport of the proteins across the mitochondrial membrane. Two candidate QT genes encoded in this small region, *Shdc* and *Tomm40l*, also localize to the mitochondria. In particular, *Tomm40l* is a channel-forming protein implicated in the import of protein precursors into mitochondria and interacts with mitochondrial targeting sequences [47]. Interestingly, the genes with transit sequences have *trans*-eQTL that map within 200 kb of *Tomm40l*, suggesting that this gene might in fact be causal for the eQTL mapping to this sub-region.

Amino acid-tRNA synthetases (ARS) are also significantly enriched among *Qrr1* target genes [25], but at a different locus. A locus at 173.3 Mb appears to be particularly important for amino acid metabolism, as seven ARS, four amino acid transporters and three other genes involved in amino acid biosynthesis map to it. Mohzui et al. [25] proposed a tRNA cluster in *Qrr1* as a functionally pertinent candidate for the tRNA synthetases. However, in BXD almost all ARS transcripts map to the distal part of *Qrr1*, where very few tRNAs can be found. Using the MDP data, the ARS genes map within 300 kb of the major tRNA cluster in *Qrr1* (25 tRNAs between 172.95 and 173.05 Mb, reported in the tRNAscan-SE Genomic tRNA database [48]). As mentioned above, genes mapping near 173.2 Mb are also enriched among *Usf1* targets. They include four ARS (*Aars*, *Cars, Yars, Mars*), two transferases (*Dpm1, B4Galt3*), an amino acid transporter (*Slc6a20a*) and an amino acid synthetase (*Asns*). Two of the genes – *B4Galt3* and *Ppox* – are also genes with *cis*-eQTL in *Qrr1*. These observations imply that either the tRNAs or *Usf1* (or both) might be causal for the expression variation among this set of amino acid metabolism related genes.

Another coherent set of eQTL could be observed for potassium transporters in the short interval from *Atp1a2* to *Kcnj10* (174.2–174.3 Mb). This locus has been associated with seizure susceptibility (*Szs1*) [49] and the two potassium channel genes encoded there – *Kcnj9* and *Kcnj10* – were proposed as primary candidates. The MDP data revealed that nearly all potassium transporters in our set map precisely to this locus - *Kcnj9* and *Kcnj10* as *cis*-eQTLs, *Kcnv* and *Kctd4* as *trans*-eQTLs. This underlines the importance of the *Szs1* locus for fine control of potassium ion homeostasis within the hippocampus and CNS tissues in general.

### *Rgs7* might act in trans inside *Qrr1*

As opposed to many other candidate regulators in *Qrr1*, *Rgs7* (regulator of G-protein signaling 7) has several target genes mapping to its promoter (177.42–177.53 Mb) and not in its transcribed region (Figure 5a). RGS proteins are important regulators of G-protein mediated signal transduction. *Rgs7* is predominantly expressed in the brain and has been found to shuttle between the cytoplasmic membrane and the nucleus, thus playing a role in gene expression in response to external stimuli [50]. Therefore, it is likely that *Rgs7* influences the expression of genes belonging to diverse functional categories. Although we could not discern a single functional class of genes *trans*-regulated by *Rgs7*, our data suggests that *Rgs7* is a master regulator in this region controlling other genes encoded in *Qrr1* in *trans* (Table S1). These genes have various functions and include *Ndufs2* (a mitochondrial NADH dehydrogenase), *Wdr42a* (a scaffolding protein expressed in the brain) and *Pex19* (a protein necessary for early peroxisomal biogenesis). Besides being targets of eQTL linking to *Rgs7*, *Wdr42a* and *Pex19* also have strong local eQTL in both BXD and MDP. The fact that some of the genes proposed as candidate regulators in *Qrr1* are simultaneously *trans*-regulated by *Rgs7* may suggest a complex, multi-layered regulatory landscape in *Qrr1* where *Rgs7* could be involved in fine tuning of the main transcriptional response mediated by those genes.

### Conclusions

Integration of low- and high-resolution eQTL data is a feasible strategy for computational fine mapping of causal genes. For a given low-resolution locus, the availability of several strong signals from a high-resolution eQTL dataset facilitates the refinement of a causal locus to specific genes.

We found several factors that influence fine mapping when using high-resolution eQTL data: the intensity of high-resolution eQTL signals, the way eQTL intervals are defined and the search/ranking scheme used and its specific parameters. Our analysis of *Qrr1* demonstrates that further integration of biological data greatly improves the overall performance of the prediction method.

Our work suggests, in accordance with previous analyses [25] that the *Qrr1 trans*-band is not caused by just one master regulator, but is due to several polymorphic genes that either independently or in a concerted way affect the expression of numerous target genes. Based on the additional power gained from our own data and because of the higher resolution of the MDP data we could identify 11 local QTL with very high confidence. The analysis of functional enrichment in sub-regions of *Qrr1* further corroborates the conclusion that *Qrr1* in fact consists of several *trans*-bands.

The MDP and BXD datasets are different in several respects. Not only are the study populations different, but the experiments were performed independently in two different labs. Because of this, our examination of consistency implicitly assesses the reproducibility of eQTL between labs. However, because the lab effect is confounded with the genetic difference of the populations we cannot distinguish these two effects. In addition, we used a different eQTL mapping method that is more suitable for mapping QTL in the MDP population [51]. We are currently working on mapping methods that are equally suitable to low- and high-density QTL data, though the results of our work show that a significant overlap between eQTL sets can already be achieved even if the mapping methods are not the same.

Bennett et al. [11] have recently published a study where they measured QTL in different RI panels and MDP lines. This certainly is the ideal situation, because the data obtained for the different populations are more consistent if measured under identical conditions in one lab. On the other hand, a very large number of physiological, behavioral and molecular QTL has already been measured for the BXD panel (see www.genenetwork.org). Our analysis demonstrates that it is possible to combine these existing data with newly obtained trait data from other populations for fine-mapping the QTL. Integrating new and existing data considerably improves the insight beyond analyzing either of the two datasets alone.

## Materials and Methods

Details about the eQTL datasets used in this study are listed in Table 1. All gene expression data were obtained from hippocampus samples using the same microarray platform (Affymetrix Mouse Genome 430 2.0). All position data in this study are set to the NCBI Mouse Genome Build 37.1 (UCSC mm9, July 2007).

### Ethics Statement

All animals received humane care according to the criteria outlined in the “Guide for the Care and Use of Laboratory Animals” prepared by the National Academy of Sciences and published by the National Institutes of Health (NIH). All animal experiments performed at GNF were approved by its Institutional Animal Care and Use Committee (IACUC). GNF's vivaria are accredited by the Association for Assessment and Accreditation of Laboratory Animal Care International (AAALAC), approval number 001051.

### MDP eQTL – Tissue Acquisition and Sample Preparation

Mice used in this study cover the following 28 inbred strains: *129S1/SvlmJ, A/J, AKR/J, BALB/cByJ, BTBR T+ tf/J, BUB/BnJ, C3H/HeJ, C57BL/6J, C57BR/cdJ, C58/J, CBA/J, CE/J, DBA/2J, FVB/NJ, I/LnJ, KK/HIJ, MA/MyJ, MRL/MpJ, NOD/LtJ, NON/LtJ, NZO/HILtJ, NZW/LacJ, P/J, PL/J, RIIIS/J, SM/J, SWR/J* and *WSB/EiJ*. Hippocampus was collected from male mice, ages 8–11 weeks, between the hours of 9 AM and noon. The anterior hippocampus was not collected. Coronal cuts were made at approximately 1.8 and 3.8 mm posterior from Bregma. A spatula was used to remove cortex and any tissue ventral to the hippocampus. Samples were immediately placed on dry ice. Frozen samples were homogenized in Trizol (Life Technologies), and resulting total RNA was cleaned using RNeasy columns (Qiagen). Total RNA from 3 mice were pooled and one microarray was used for each pool. The Affymetrix One Cycle kit was used to make cRNA from total RNA. Array hybridization, washing, and scanning followed standard Affymetrix protocols.

### eQTL Mapping

It has been reported that sequence polymorphisms in probe regions may cause many false *cis*-eQTL, as they lead to differences in hybridization, and not in gene expression [52]. A recent, high quality set of SNPs has been used to correct BXD gene expression data (about 7 million SNPs generated by sequencing C57BL/6J and DBA/2J genotype strains, using SOLiD and Solexa short sequence reads – R.Williams et al., unpublished data - www.genenetwork.org). The M430 2.0 probes in the BXD eQTL dataset containing one or more SNPs have been removed.

For the BXD panel, eQTL associations have been inferred using Haley-Knott regression [53], which is equivalent to the original publication of the BXD data [24].

MDP expression data have been corrected for SNPs in probe regions using available data at dbSNP 126 (May 2006). MDP eQTL mapping was performed using methods that have been previously described [54]. Briefly, we use ANOVA to calculate the strength of genetic associations between an input phenotype and the ancestral haplotype structure (as inferred using a local window of three adjacent SNP alleles across the genome). A weighted bootstrap method was introduced to detect association peaks conditional on the population structure in the MDP [21]. At each genetic locus, the association score (S_a_) was represented as the negative log_10_-transformed p-value. HAM analysis was performed for all differentially expressed genes across the 28 strains. Expression phenotypes were in log scale.

### eQTL Comparison Strategy

Since the MDP dataset has a higher density of informative markers than the BXD dataset, there is not a simple 1:1 relationship between the markers. Hence, two comparison schemes have been tested: exact and interval-based. The **exact scheme** only uses markers that are common to the two datasets (i.e. at common genomic positions, ±5 bp). Thus, MDP markers that are between two informative markers from the BXD cross are ignored. The **interval scheme** integrates all markers of the MDP study within some interval around the markers from the BXD population. In this case we assign the MDP markers to the closest BXD marker and compare the maximum MDP signal of those markers to the respective BXD signal. For both schemes, multiple common peaks within a 5 Mb window are only counted once [21].

We determine the significance of two matching eQTL peaks based on an empirical distribution of eQTL scores. First, we discretized the range of scores for both the MDP and BXD data (above the 99% quantile), which establishes a two-dimensional grid of the two scoring schemes. Then, for each threshold combination (i.e. each bin), a background distribution is established using 10^4^ random eQTL profile comparisons and counting the number of matching eQTL.

### Genomic Locations – Local and Distant eQTL

In order to identify local (*cis*-) eQTL and distant (*trans*-) eQTL we mapped the probe sets (Affymetrix Mouse 430 2.0) to the corresponding genomic locations (NCBI Mouse Genome Build m37.1). Local eQTL have been defined as eQTL within 5 Mb of the physical location of the gene itself [21], [22]. Using shorter intervals for defining local eQTL did not significantly change the conclusions (not shown). For the study of *Qrr1*, a smaller threshold (500 kb) has been used to define local eQTL.

To search for the presence of common co-localizing distant eQTL (eQTL hotspots, or *trans*-bands), the entire genome was divided into 2 Mb bins (1320 total bins) and the number of significant distant eQTL (in common between the MDP and BXD datasets) was counted in each bin.

### eQTL Fine Mapping Assessment

We used independent, external gene annotations to test the efficiency of our methods for fine mapping of loci to specific causal genes. This approach is based on the assumption that genes whose products participate in the same molecular pathway are also likely to influence each other's expression levels [55]. Hence, a candidate source gene of some eQTL is assumed to be correct if both the candidate and target gene are members of the same KEGG pathway (KEGG release 49.0 [56]). We ran the eQTL fine mapping for 3969 unique mouse genes being annotated as KEGG pathway members.

### Selection of Significant eQTL Intervals for the Fine Mapping Analysis

For every marker *m_i_* within the low-resolution eQTL dataset (BXD), we defined its associated eQTL interval as the genomic region delimited by the flanking markers (*m_i−1_, m_i+1_*). For assessing the performance of MDP-based fine mapping, we decided to restrict the analysis to intervals with strong association signals in both the BXD and MDP datasets. Thus, we selected eQTL intervals matching all of the following criteria:

1. BXD eQTL signal ≥99.99% quantile in BXD eQTL dataset, genome-wide;
2. MDP eQTL signal ≥99.99% quantile in MDP eQTL dataset, genome-wide;
3. Number of genes within the eQTL region >1;
4. Number of MDP (high resolution) eQTL signals >1;
5. The target gene is annotated as a member of at least one KEGG pathway;
6. At least one gene from the selected eQTL interval is a member of the same KEGG pathway.

The first two criteria ensure that the loci contain high BXD and MDP signals. Using MDP data for fine mapping does not make sense if criteria 3 and 4 are not fulfilled. The last two criteria restrict the analysis to eQTL with sufficient prior knowledge. The final selection included 105 eQTL intervals that confirmed with the above criteria and hence could be used for subsequent analysis.

### Scoring Candidate Genes

We reformulate the fine-mapping task as follows: Given a genomic interval around a marker that is significant according to the BXD data, the task is to rank the genes within this interval based on the MDP data. We then evaluate the ranking based on the known pathway memberships. We assigned a score to each gene in the interval based on the MDP scores as follows:

- If there is at least one marker in the transcribed region of the gene, use the maximum of those markers.
- If there is no marker in the transcribed region, use the maximum score of the flanking markers. The genomic region that is searched in this case extends symmetrically downstream and upstream the transcribed region and is defined by a fixed length in bp (specified by the parameter ‘*ext*’).

Subsequently, genes in each search interval were ranked based on those MDP scores and the top ranking gene was selected as the predicted causal gene. If this top-ranking gene is in a common KEGG pathway with the target gene, the ranking is called a ‘success’. We then compute the fraction of correct predictions among all *N* intervals assessed, *S_HR_* = *N_successes_*/*N*, where *N_successes_* is the number of correct predictions.

This score is then compared to:

- the expected random success rate, which is the average frequency of “true” causal genes in the *N* cases: 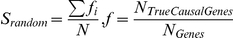;
- the success rate *S_closest_* obtained by selecting the closest gene to the low-resolution (BXD) marker.

### Use of Genetic Distances to Narrow Locus Sizes

We used genetic distances from Mouse HS genetic map-Build 37 available at the Wellcome Trust Center for Human Genetics (http://www.well.ox.ac.uk/mouse). Mapping of MDP known markers to the HS genetic map provides 9,843 markers with genetic distances. We then used this information to specify eQTL intervals in terms of genetic distances (ΔcM) instead of base pairs, in order to restrict interval sizes (and number of genes) associated with each selected eQTL. When genetic distance was not available for the interval under study, we assumed this parameter to vary linearly with physical distance for small intra-marker regions (typically few hundred kb). Hence, we retrieved cM value by means of linear interpolation between the two closest markers with known genetic distance.

### Usf1 and Atf6 Over-expression Datasets

Plaisier et al. [44] studied overexpression of *Usf1* in HEK293T cells *in vitro* to ascertain the genes downstream of *Usf1* (GEO accession: GSE17300). CEL files from the repository were processed as described in [44]. Differential expression between *Usf1* and empty vector control transfected cells was analyzed using a Student's t-test. Differentially expressed genes were selected at different p-value cutoffs (0.1, 0.05, 0.001); p≤0.05 was used for the enrichment illustrated in Fig. 5c; additional cutoffs are reported in Figure S4. Human genes were mapped to murine genes using the available orthology data at Mouse Genome Informatics (MGI 4.32, December 2009; http://www.informatics.jax.org).

For *Atf6*, overexpression was studied in mouse heart tissue [46] (GEO accession: GSE8311). We selected 607 genes exhibiting at least 2-fold differential expression (p≤0.001).

### Analysis of Tissue Specificity in Gene Expression

The GNF Mouse GeneAtlas V3 was used as an independent source of information about tissue-specific gene expression. In this dataset, 78 tissues were taken from naïve male C57BL6 mice for hybridization to MOE 430-2 arrays [57] (https://biogps.gnf.org). CNS-specific gene expression was evaluated by testing the distribution of expression levels in the CNS (11 tissues) against the distribution of expression levels across all tissues included in GeneAtlas (two-sided Student's t-test). Tissue-specific expression levels were averaged in case of genes targeted by multiple probesets.

### Functional Enrichment Analysis

Enrichment in functional categories was evaluated using the analytical tool DAVID 6.7 (http://david.abcc.ncifcrf.gov) [58]. Overrepresented functional terms were identified and statistical significance of enrichment was calculated using a modified Fisher's Exact Test or EASE score [59].

## Supporting Information

Table S1 Regulators (quantitative trait genes) for Qrr1 as suggested by MDP mapping data.
(0.05 MB DOC)

Table S2 Functional enrichment analysis of genes that map to Qrr1 in the BXD hippocampus data set.
(0.02 MB XLS)

Figure S1 Assessment of MDP-based candidate gene selection - *trans*-eQTL only. For each of the selected loci a selected candidate gene was considered as a true positive if it is part of a common pathway with the target gene. The figure shows the fraction of loci with correct selections; x-axis shows inclusion of successively more eQTL intervals at decreasing MDP scores (between top 100 and top 300 strongest signals). Black line: true positive rate (TPR) using MDP signals. Blue line: TPR using the closest gene. Solid red line: TPR using random gene selection (average across 100 randomizations). Dotted red lines: average TPR for random gene selection ± standard deviation. We also confirmed that genes being part of the same pathway rank significantly higher when using the MDP data for fine-mapping (analysis equivalent to Figure 3A). We focused on the top 200 intervals without *cis*-eQTL. Candidate genes from the same pathway were ranked among the top 50% in 87 cases and among the bottom 50% in 67 cases (46 cases not ranked; difference is significant with p<10^−16^, one-sided t-test).
(0.00 MB PDF)

Figure S2 Correlation between the strength of MDP eQTL scores and their ability to correctly map causal genes. The eQTL intervals are ordered according to decreasing MDP eQTL scores, e.g. longer lists include lower scores.
(0.00 MB PDF)

Figure S3 MDP-based candidate gene selection at different ΔcM.
(0.13 MB PDF)

Figure S4 Enrichment of Usf1 targets along Qrr1 at different thresholds in the BXD and MDP datasets, and different p-value cutoffs for defining targets from the over-expression experiment. A: lower p-value cutoff (0.1, 5839 targets); B: higher p-value cutoff (0.01, 850 targets); C: lower BXD threshold (99.5% percentile, 285 genes selected); D: higher MDP threshold (99.9% percentile, 37 genes selected). E: Enrichment of Atf6 targets along Qrr1. Black line: enrichment (-logp) as a function of the physical distance from the transcription factor, i.e. the length of an exploratory interval centred on Atf6. Red lines show results for randomized Atf6 targets (average across 100 randomizations ± standard deviation).
(0.39 MB PDF)

Figure S5 Functional enrichment of target genes linking to Qrr1. The average MDP eQTL score at each marker in Qrr1 is reported for all genes of a specific class.
(0.84 MB PDF)
