# Supplementary material for: Integrative Analysis of Low- and High-Resolution eQTL
Source: PLoS One. 2010 Nov 10;5(11):e13920. doi: 10.1371/journal.pone.0013920 (PMC2978079; doi:10.1371/journal.pone.0013920)
Supplement: Table S1 — Regulators (quantitative trait genes) for Qrr1 as suggested by MDP mapping data. (0.05 MB DOC) [file pone.0013920.s001.doc]

**Supplementary Table S1**: Regulators (quantitative trait genes) for *Qrr1* as suggested by MDP mapping data.

| **Predicted QT Gene** | **Target** | **Link Type** |
| --- | --- | --- |
| **Apoa2 (closest cis: Fcer1g, 3kb)** | Mllt11 |  |
| **Apoa2 (closest cis: Fcer1g, 3kb)** | Pqbp1 |  |
| **B4galt3** | Mrpl50 |  |
| **Cadm3 (closest cis: Darc, 3kb)** | Rnf6 |  |
| **(Cadm3)** | Darc | *B* |
| **(Casq1)** | Wdr42a | *B* |
| **(Copa)** | Pex19 | *B* |
| **Hsd17b7 *** | Sfrs3 |  |
| **Hsd17b7 *** | 2700007P21Rik |  |
| **Hsd17b7 *** | Eif4g2 |  |
| **Fcgr2b ( closest cis: Shdc, 150kb)** | Hnrpab |  |
| **Fmn2** | Fmn2 | *A* |
| **Fmn2** | Gas2l1 |  |
| **Fmn2** | Snrpd3 |  |
| **Grem2** | Slc7a1 |  |
| **Kcnj9** | Kcnj9 | *A* |
| **Kcnj10** | Kcnj10 | *A* |
| **Klhdc9** | Cars |  |
| **(Ly9)** | Ppox | *B* |
| **Ndufs2** | 2500003M10Rik |  |
| **Olfr220 (closest cis: Fmn2, 50Kb)** | 9530048O09Rik |  |
| **Pbx1 (cis in BXD/MDP)*** | Rbmx |  |
| **Pcp4l1** | Wdr74 |  |
| **Pcp4l1** | 4933433P14Rik |  |
| **(Pex19)** | Nit1 | *B* |
| **Pfdn2** | Mars |  |
| **(Pfdn2)** | B4galt3 | *B* |
| **Rgs7** | Ndufs2 |  |
| **Rgs7** | Itpka |  |
| **Rgs7** | Dgki |  |
| **Rgs7** | Atp5j2 |  |
| **Rgs7** | Lrrtm1 |  |
| **Rgs7** | Ilf2 |  |
| **Rgs7** | Pex19 | *C* |
| **Rgs7** | Wdr42a | *C* |
| **Sdhc** | Sdhc | *A* |
| **(Slamf1)** | Copa | *B* |

**Link Types:**

**A:** Confirmed *cis*-eQTL with maximum MDP score at the gene

**B:** Putative *cis*-eQTL with maximum MDP score above different gene, but close to the target gene (< 500 kb). Gene with highest MDP eQTL is reported in the column on the left.

**C:** Putative local *trans*-eQTL, *i.e*. source and target are both in Qrr1. The MDP data suggests that the QT gene is different from the target gene.
