# Supplementary figures and images for: Integrative Analysis of Low- and High-Resolution eQTL
Source: PLoS One. 2010 Nov 10;5(11):e13920. doi: 10.1371/journal.pone.0013920 (PMC2978079; doi:10.1371/journal.pone.0013920)

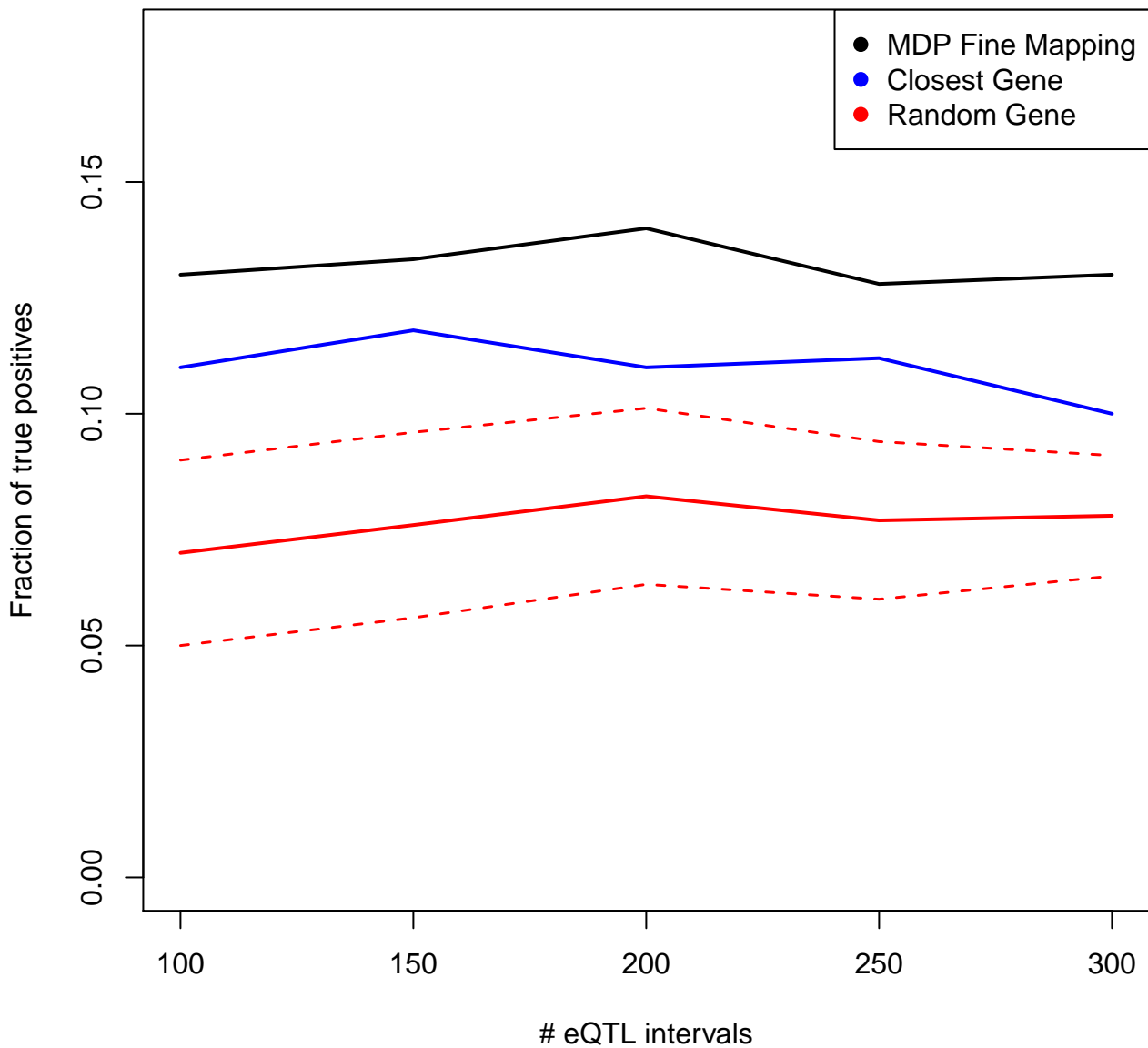

Supplement: Figure S1 — Assessment of MDP-based candidate gene selection - trans-eQTL only. For each of the selected loci a selected candidate gene was considered as a true positive if it is part of a common pathway with the target gene. The figure shows the fraction of loci with correct selections; x-axis shows inclusion of successively more eQTL intervals at decreasing MDP scores (between top 100 and top 300 strongest signals). Black line: true positive rate (TPR) using MDP signals. Blue line: TPR using the closest gene. Solid red line: TPR using random gene selection (average across 100 randomizations). Dotted red lines: average TPR for random gene selection ± standard deviation. We also confirmed that genes being part of the same pathway rank significantly higher when using the MDP data for fine-mapping (analysis equivalent to Figure 3A). We focused on the top 200 intervals without cis-eQTL. Candidate genes from the same pathway were ranked among the top 50% in 87 cases and among the bottom 50% in 67 cases (46 cases not ranked; difference is significant with p<10−16, one-sided t-test). (0.00 MB PDF) [file pone.0013920.s003.pdf]

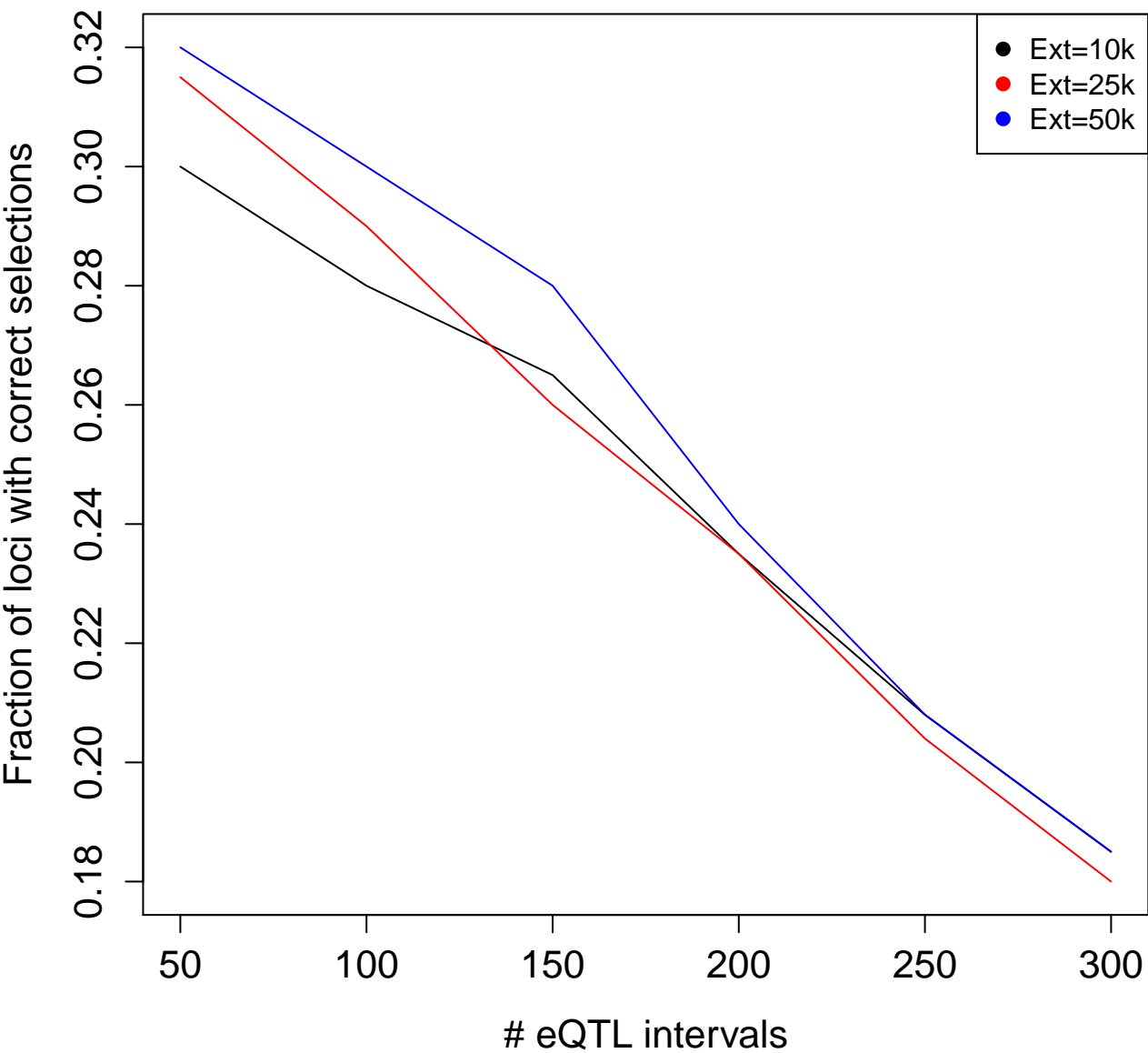

Supplement: Figure S2 — Correlation between the strength of MDP eQTL scores and their ability to correctly map causal genes. The eQTL intervals are ordered according to decreasing MDP eQTL scores, e.g. longer lists include lower scores. (0.00 MB PDF) [file pone.0013920.s004.pdf]

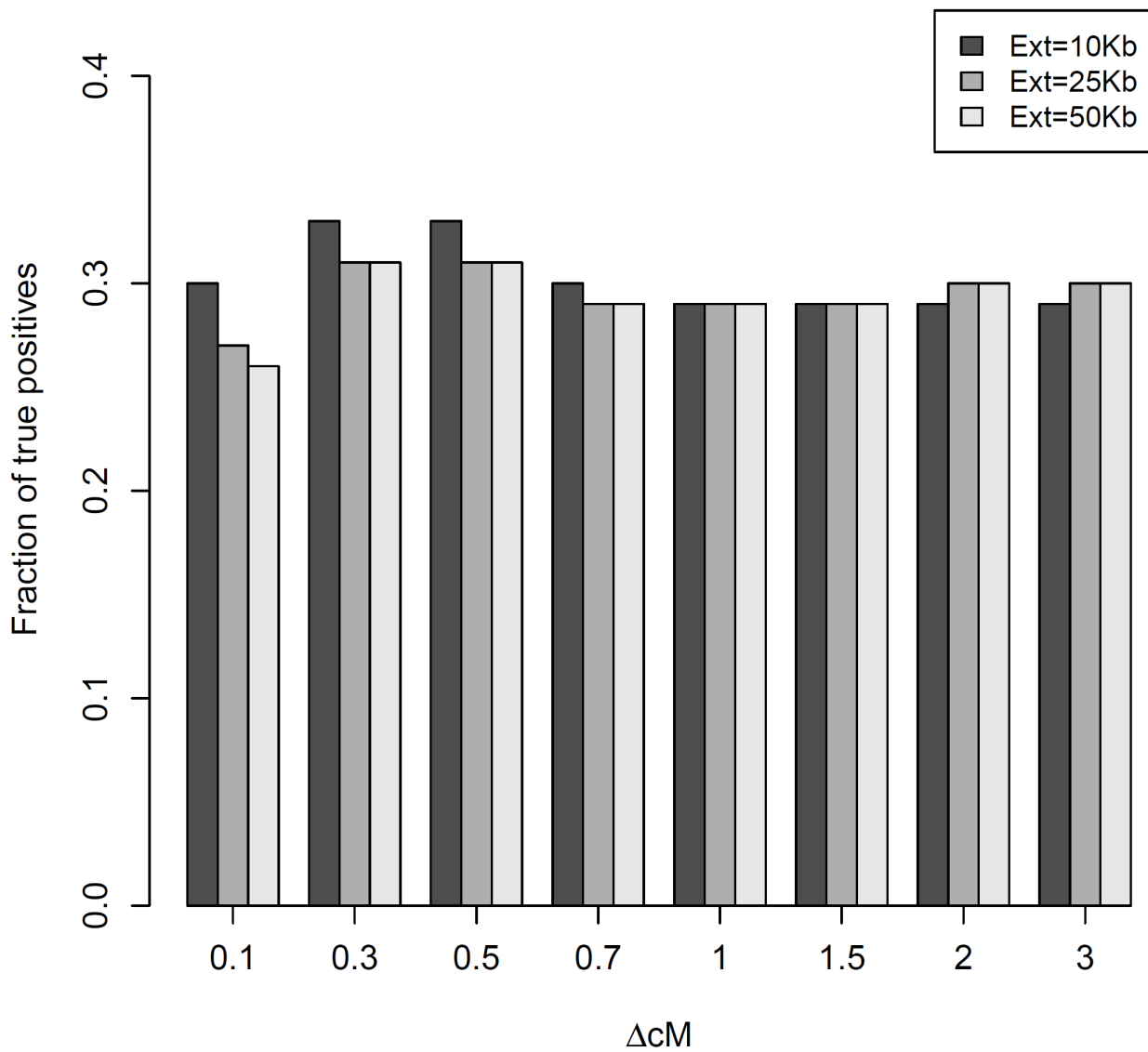

Supplement: Figure S3 — MDP-based candidate gene selection at different ΔcM. (0.13 MB PDF) [file pone.0013920.s005.pdf]

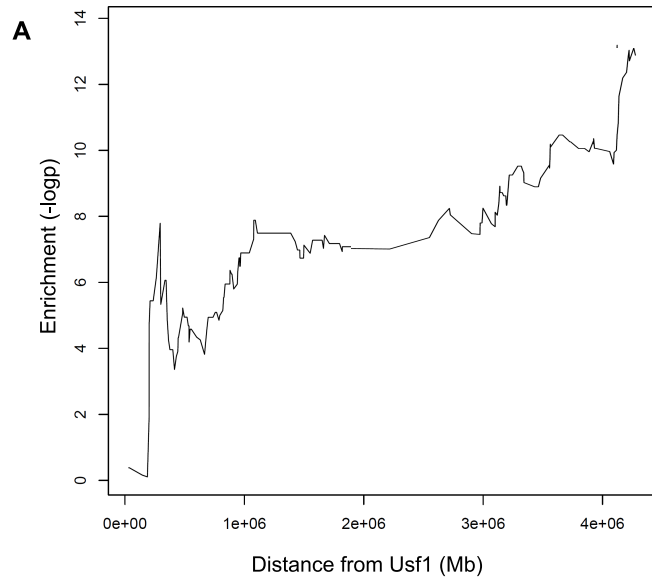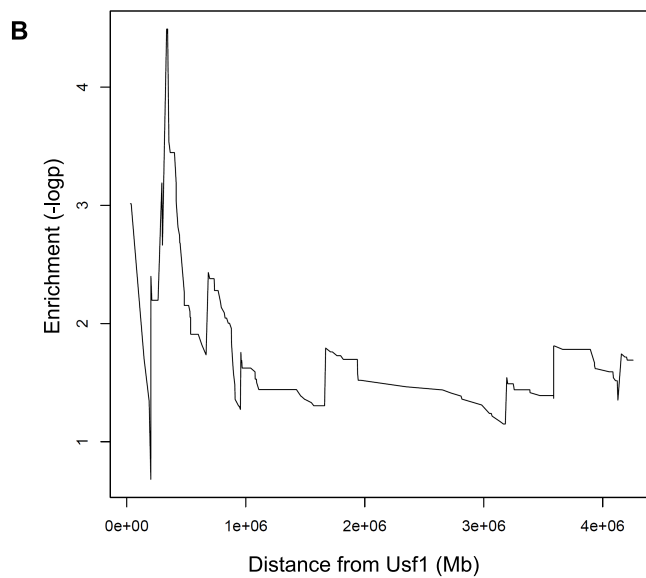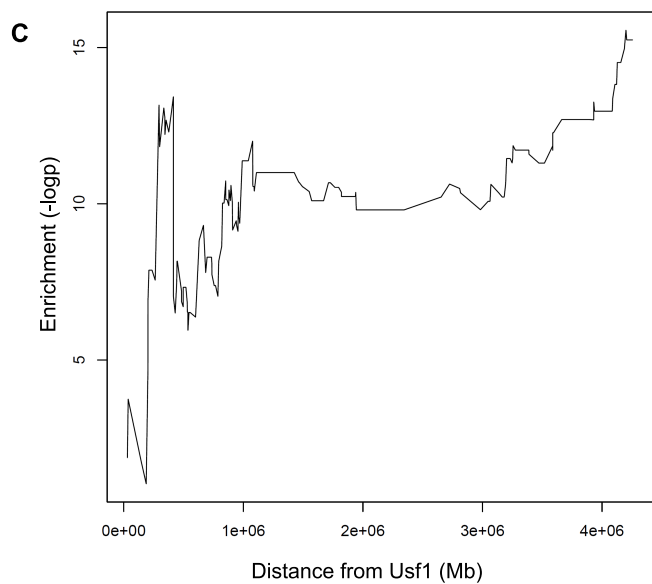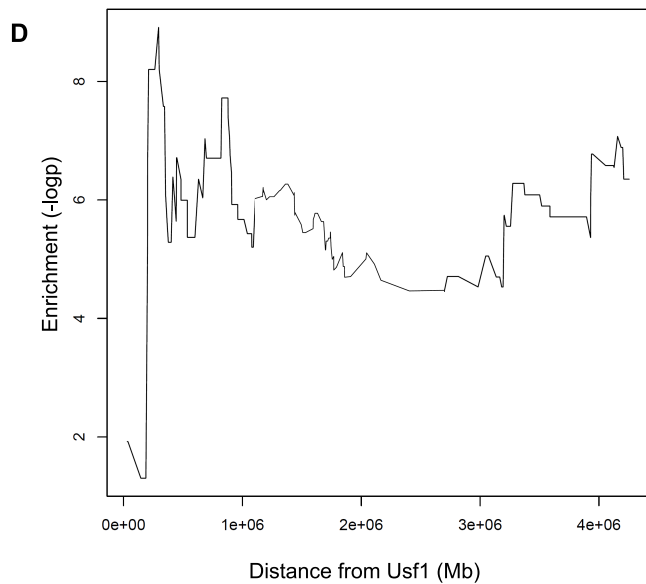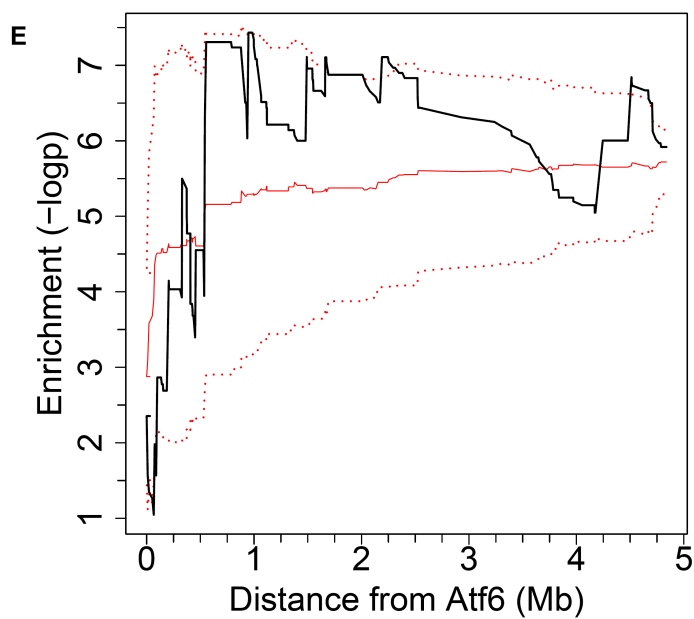

Supplement: Figure S4 — Enrichment of Usf1 targets along Qrr1 at different thresholds in the BXD and MDP datasets, and different p-value cutoffs for defining targets from the over-expression experiment. A: lower p-value cutoff (0.1, 5839 targets); B: higher p-value cutoff (0.01, 850 targets); C: lower BXD threshold (99.5% percentile, 285 genes selected); D: higher MDP threshold (99.9% percentile, 37 genes selected). E: Enrichment of Atf6 targets along Qrr1. Black line: enrichment (-logp) as a function of the physical distance from the transcription factor, i.e. the length of an exploratory interval centred on Atf6. Red lines show results for randomized Atf6 targets (average across 100 randomizations ± standard deviation). (0.39 MB PDF) [file pone.0013920.s006.pdf]
