# Supplementary material for: Integrative Analysis of Low- and High-Resolution eQTL
Source: PLoS One. 2010 Nov 10;5(11):e13920. doi: 10.1371/journal.pone.0013920 (PMC2978079; doi:10.1371/journal.pone.0013920)
Supplement: Figure S5 — Functional enrichment of target genes linking to Qrr1. The average MDP eQTL score at each marker in Qrr1 is reported for all genes of a specific class. (0.84 MB PDF) [file pone.0013920.s007.pdf]

Amino-acid synthetases

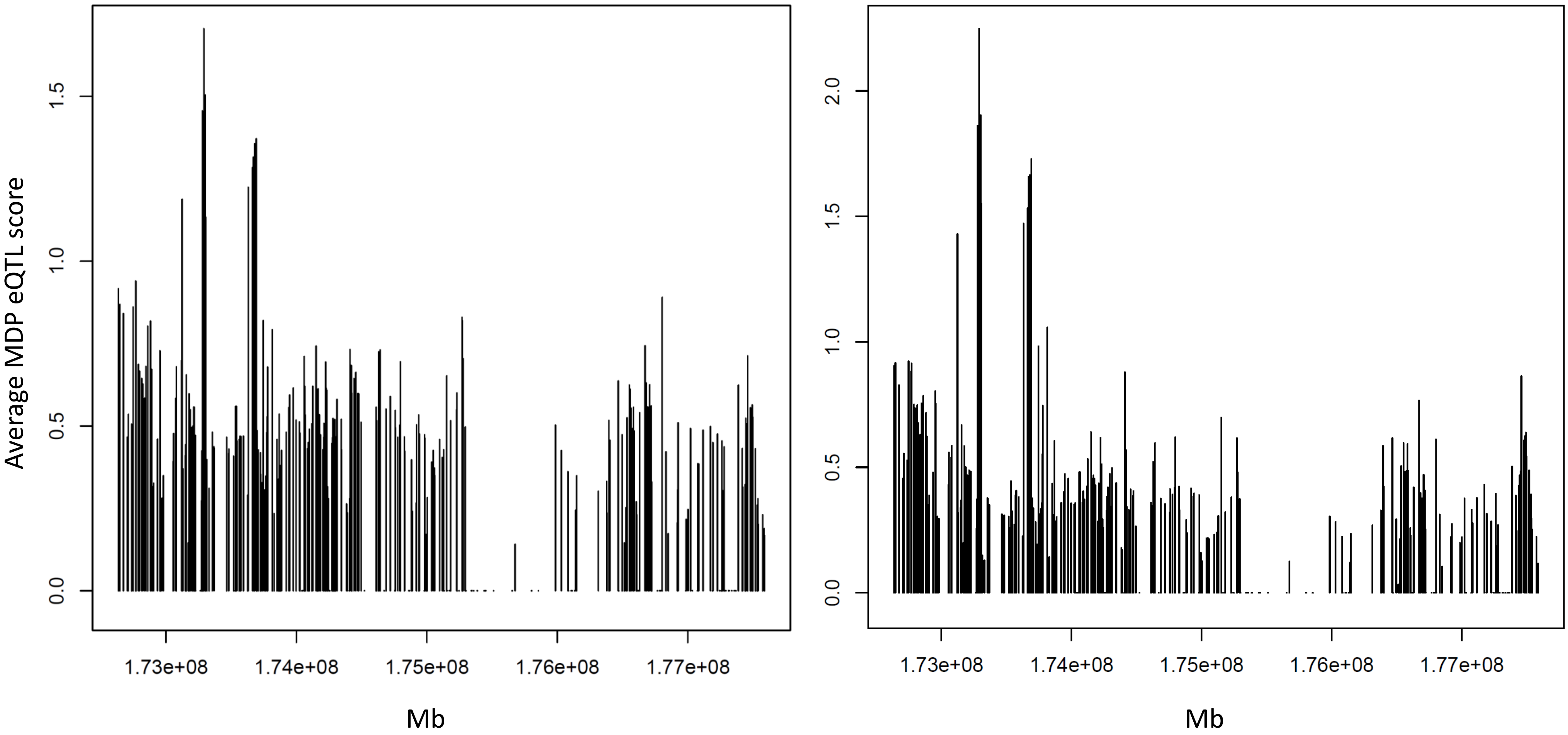

Significant in BXD: 7 genes

Significant in BXD and MDP: 5 genes

Amino-acid transporter

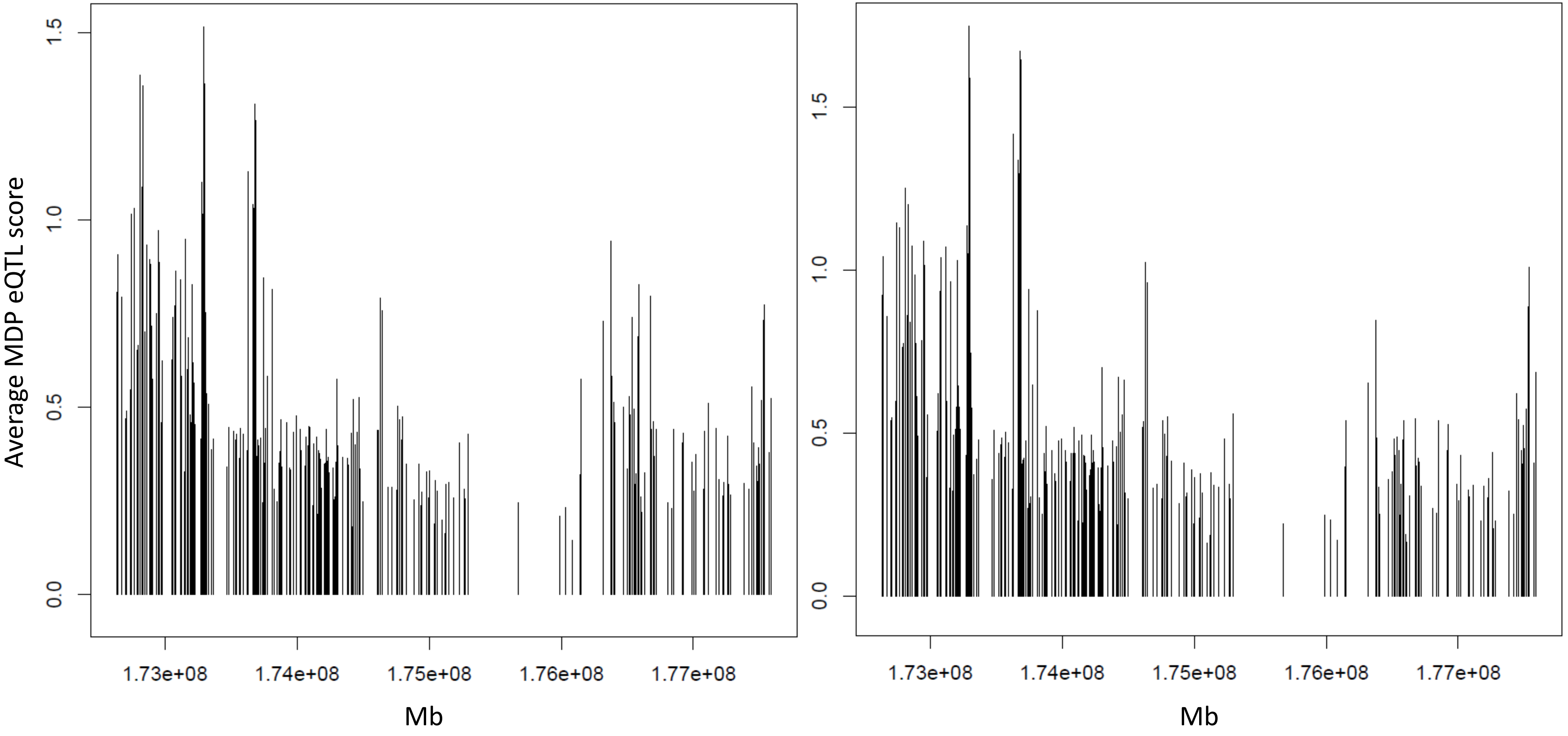

Significant in BXD: 4 genes

Significant in BXD and MDP: 3 genes

Amino-acid biosynthesis

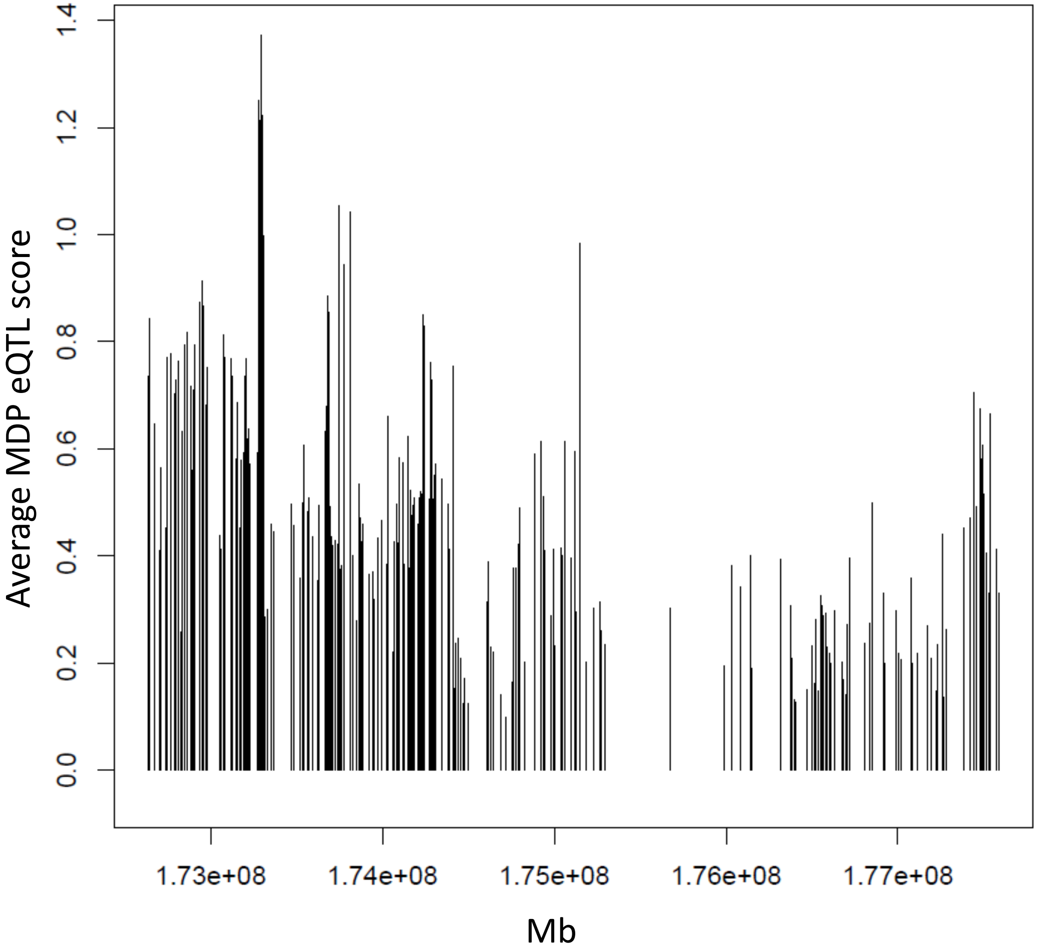

Significant in BXD: 3 genes

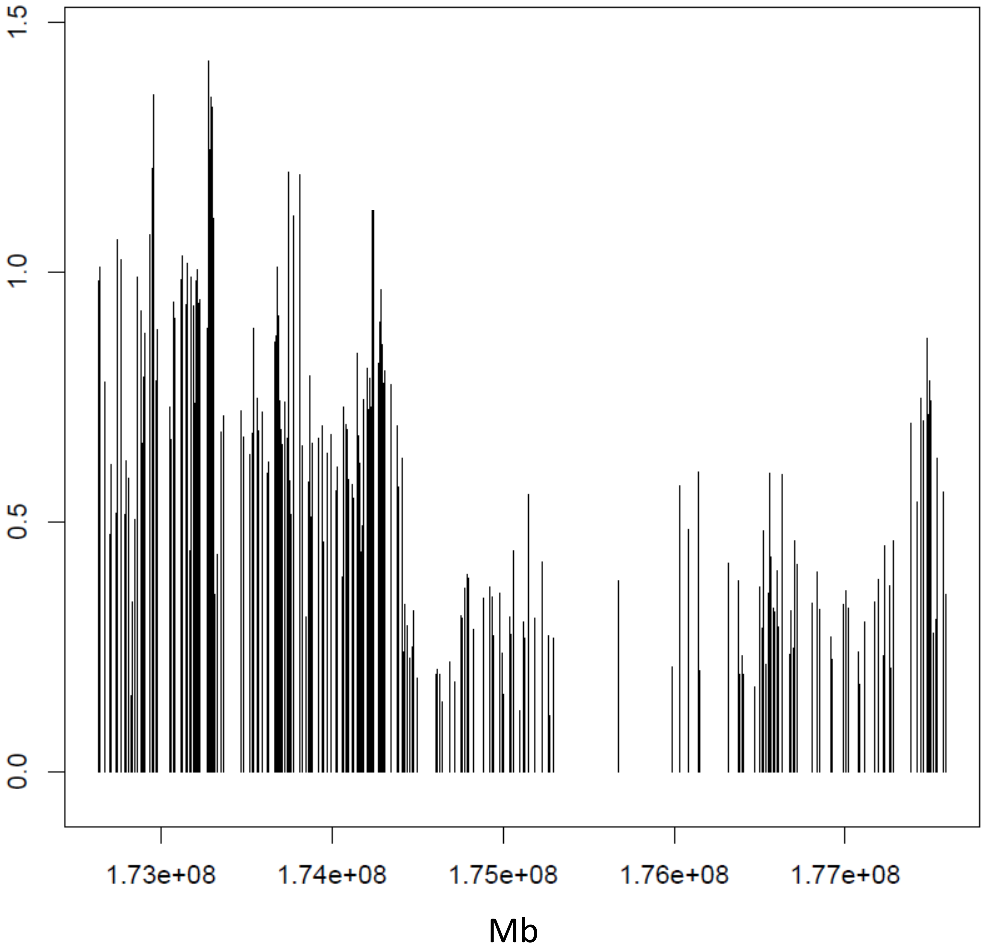

Significant in BXD and MDP: 2 genes

Mitochondrion

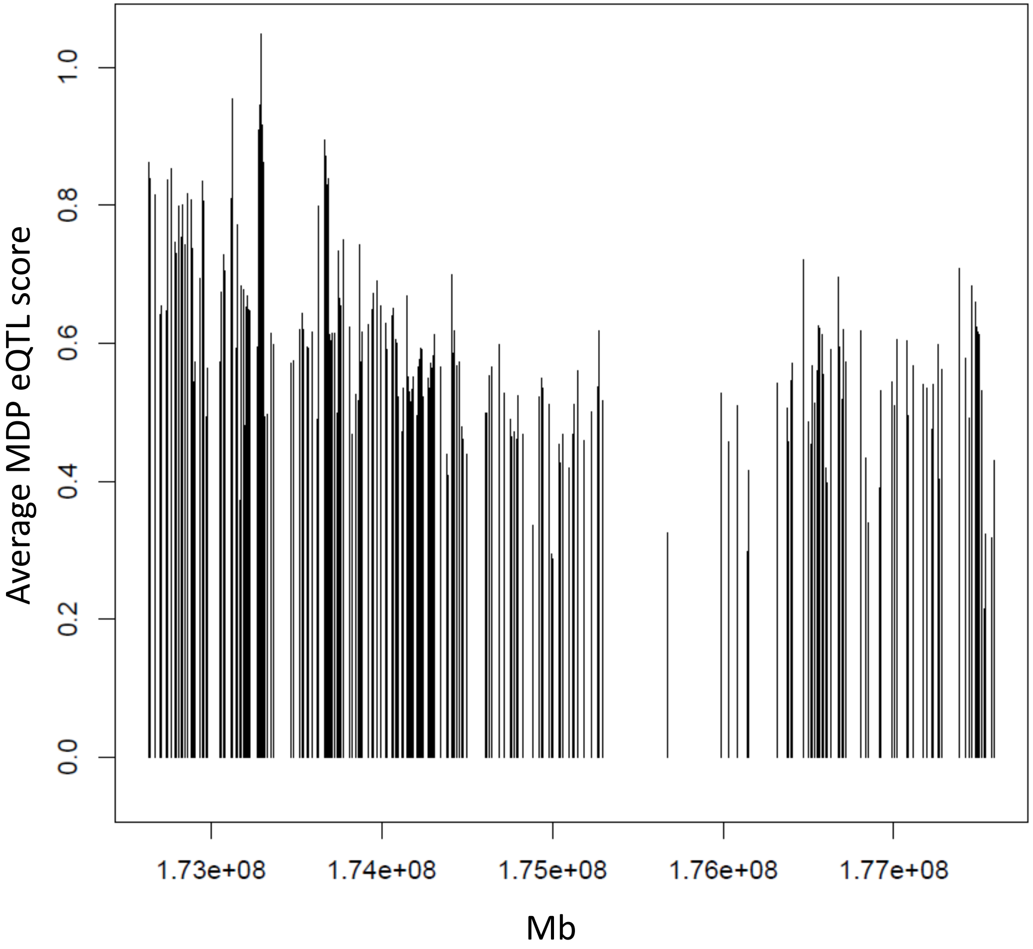

Significant in BXD: 26 genes

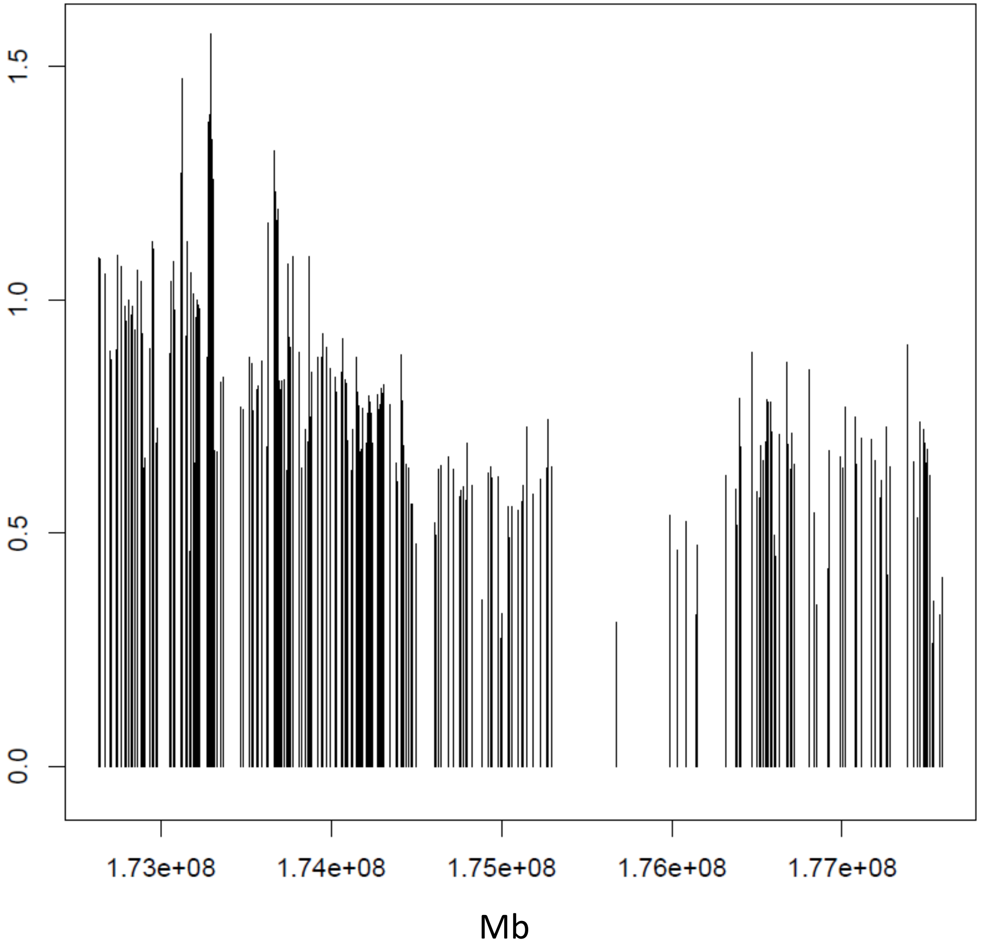

Significant in BXD and MDP: 11 genes

Transit Peptide

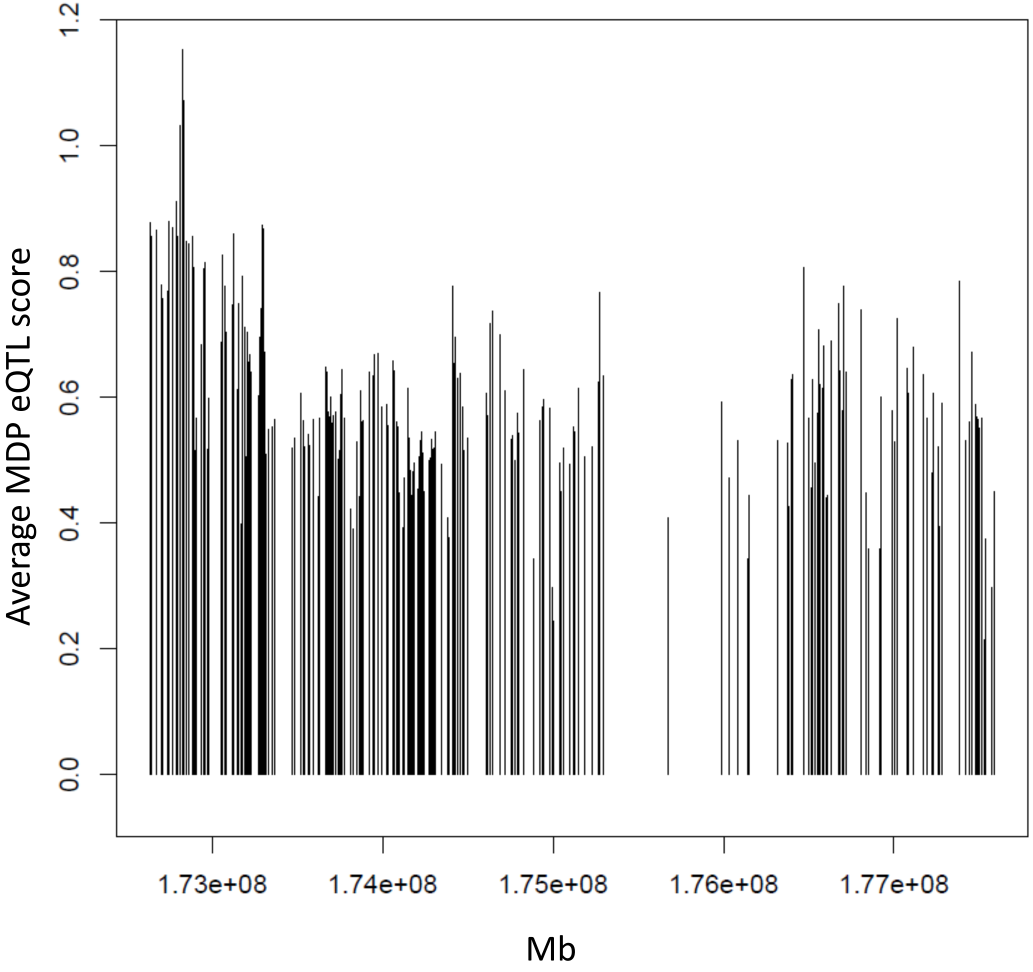

Significant in BXD: 10 genes

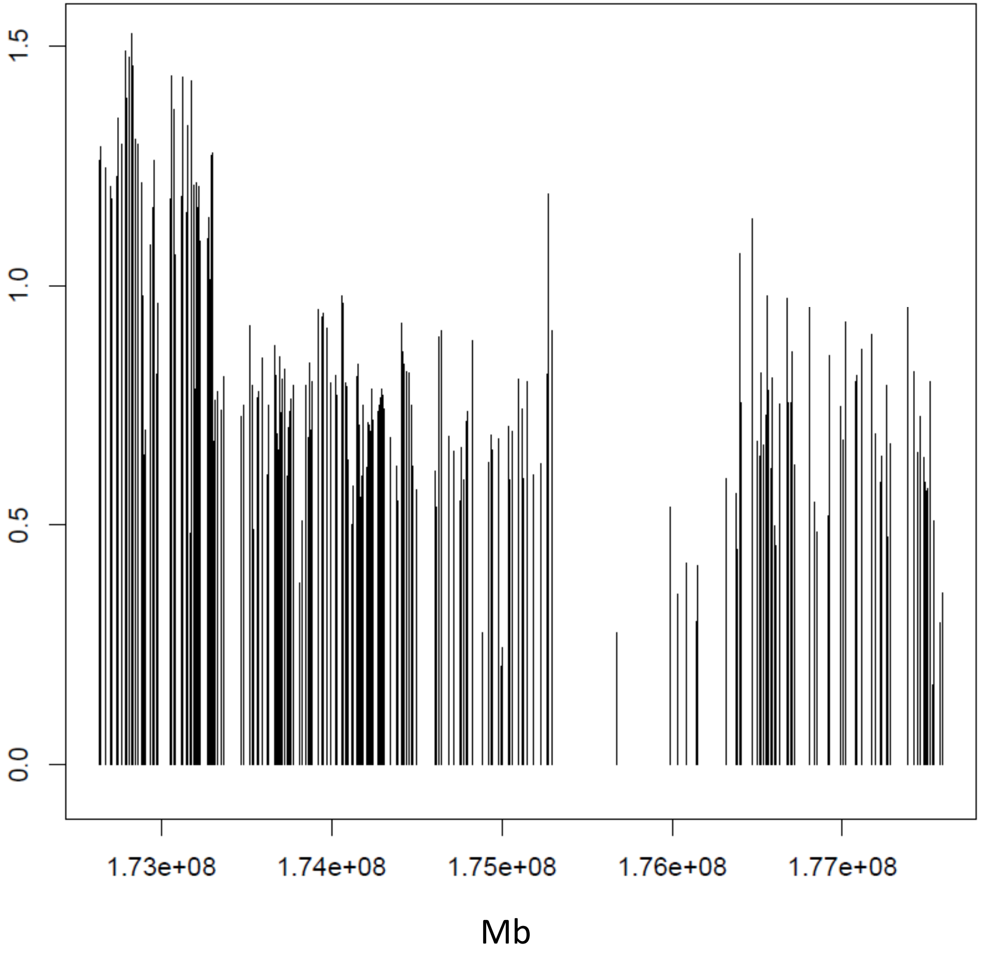

Significant in BXD and MDP: 5 genes

Potassium Ion Transport

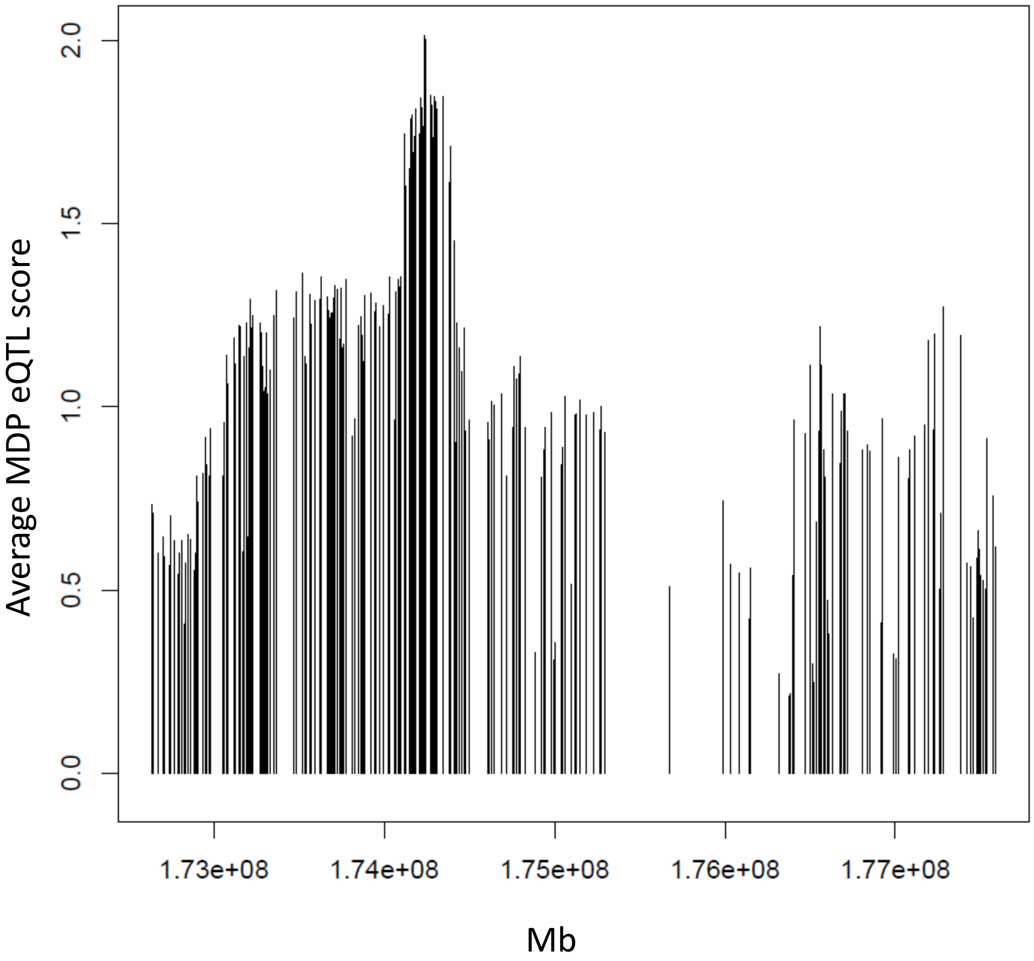

Significant in BXD: 4 genes

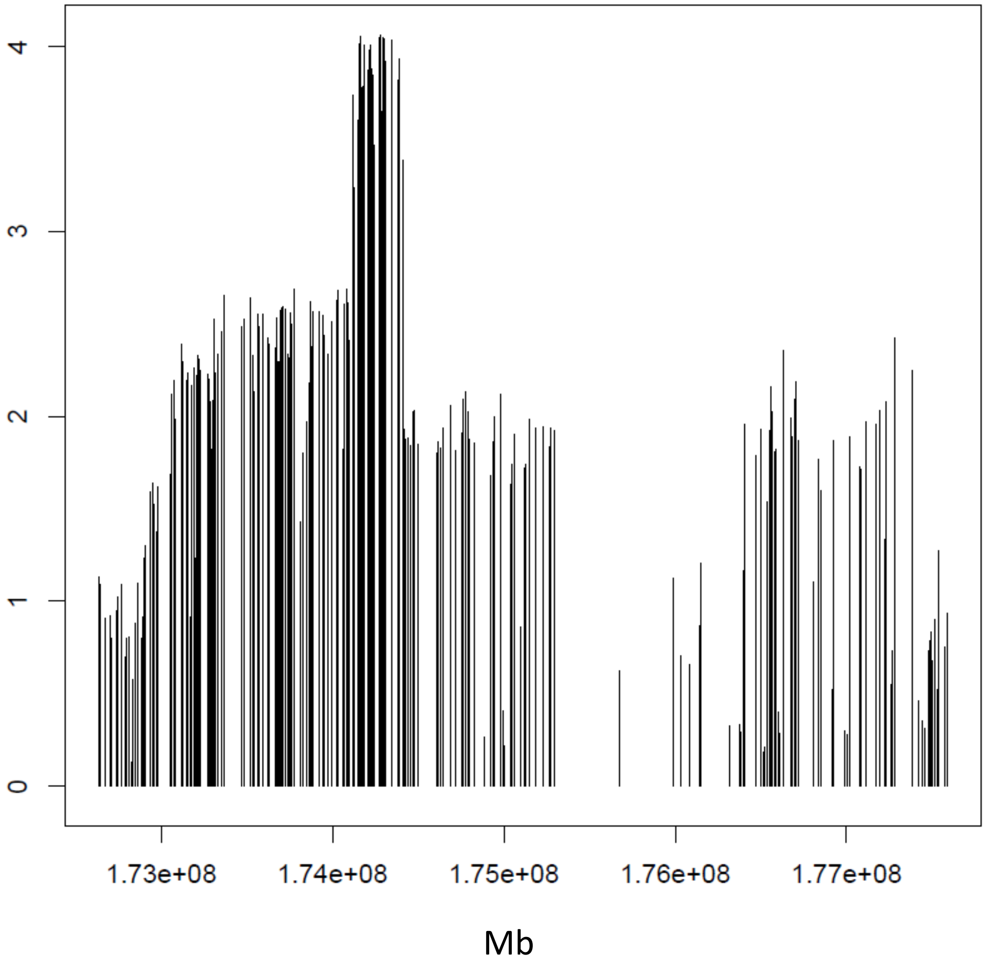

Significant in BXD and MDP: 2 genes

Neuron Projection

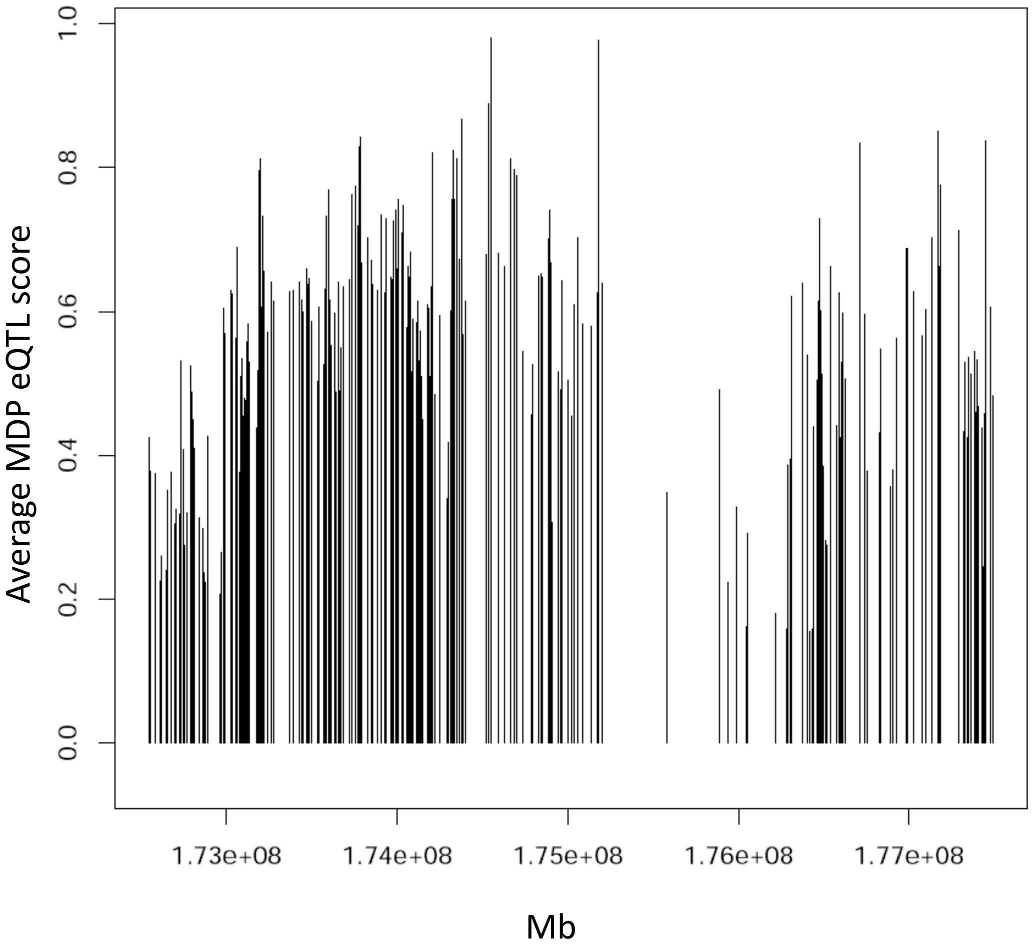

Significant in BXD: 5 genes

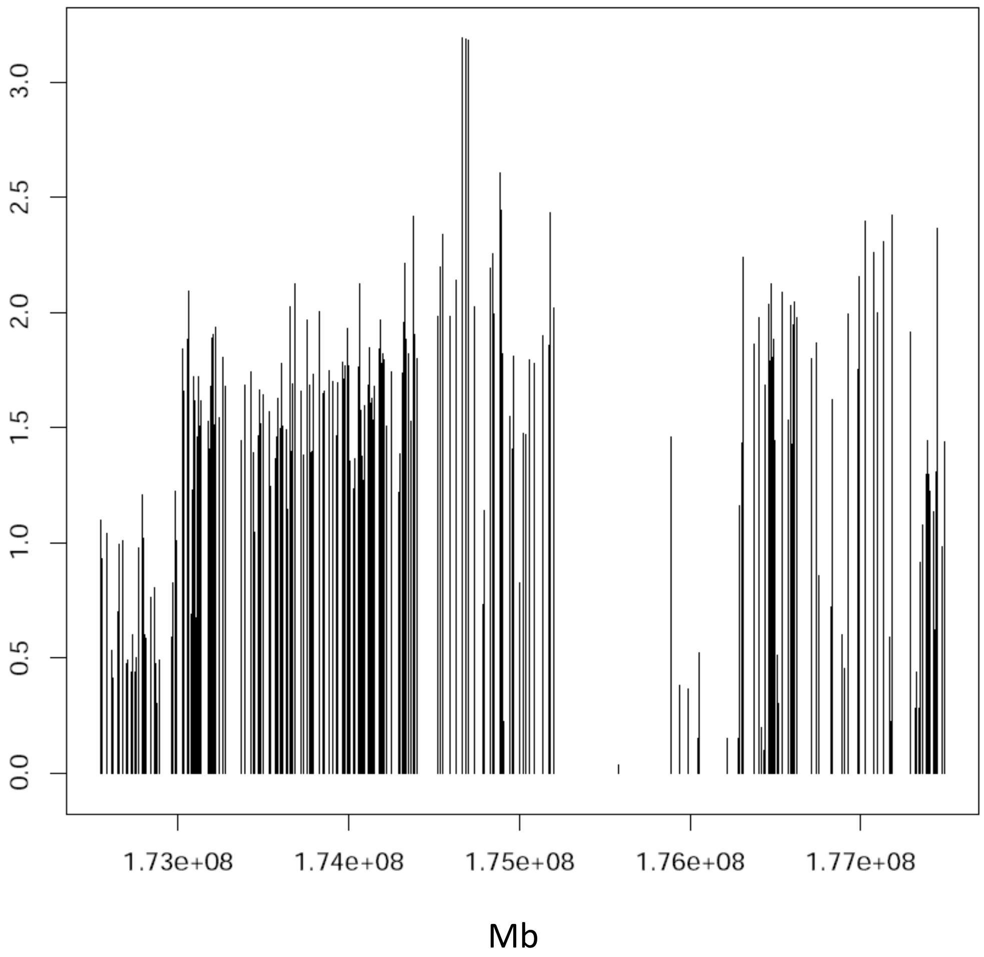

Significant in BXD and MDP: 2 genes

RAB Proteins

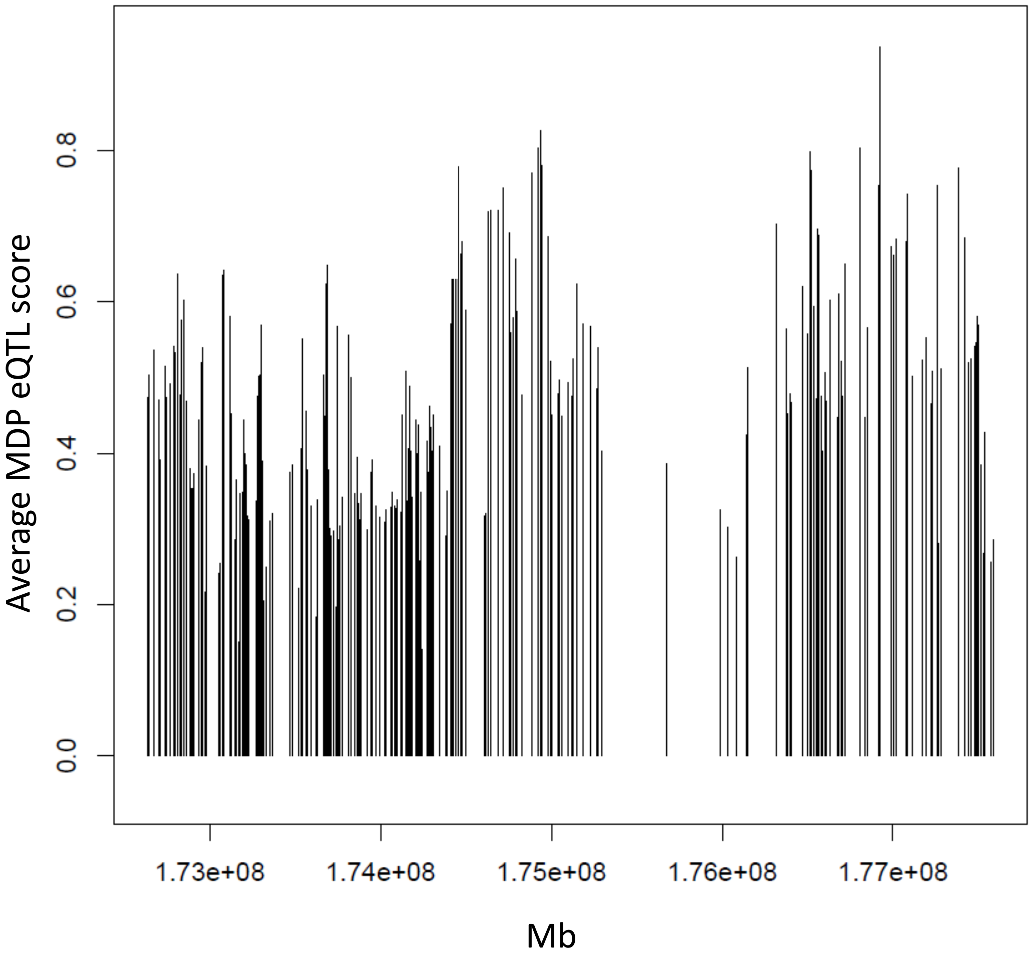

Significant in BXD: 3 genes

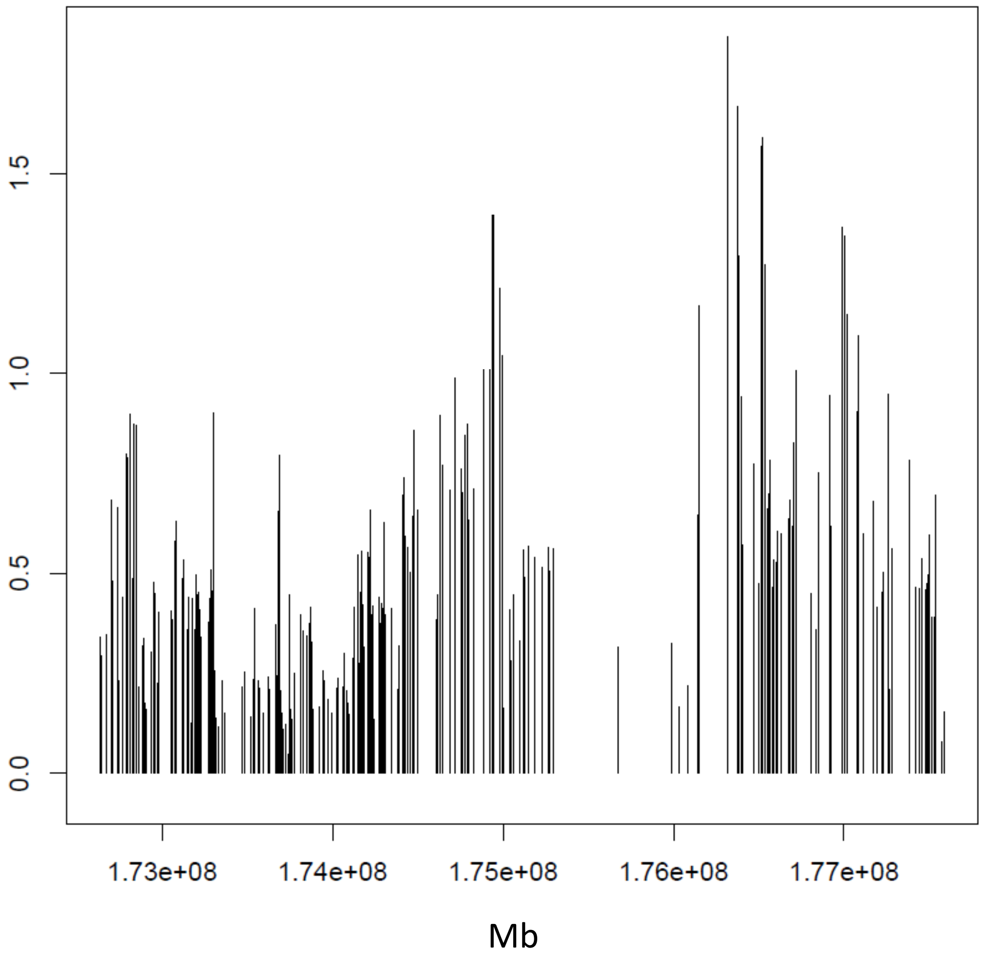

Significant in BXD and MDP: 2 genes

WD40 repeat proteins

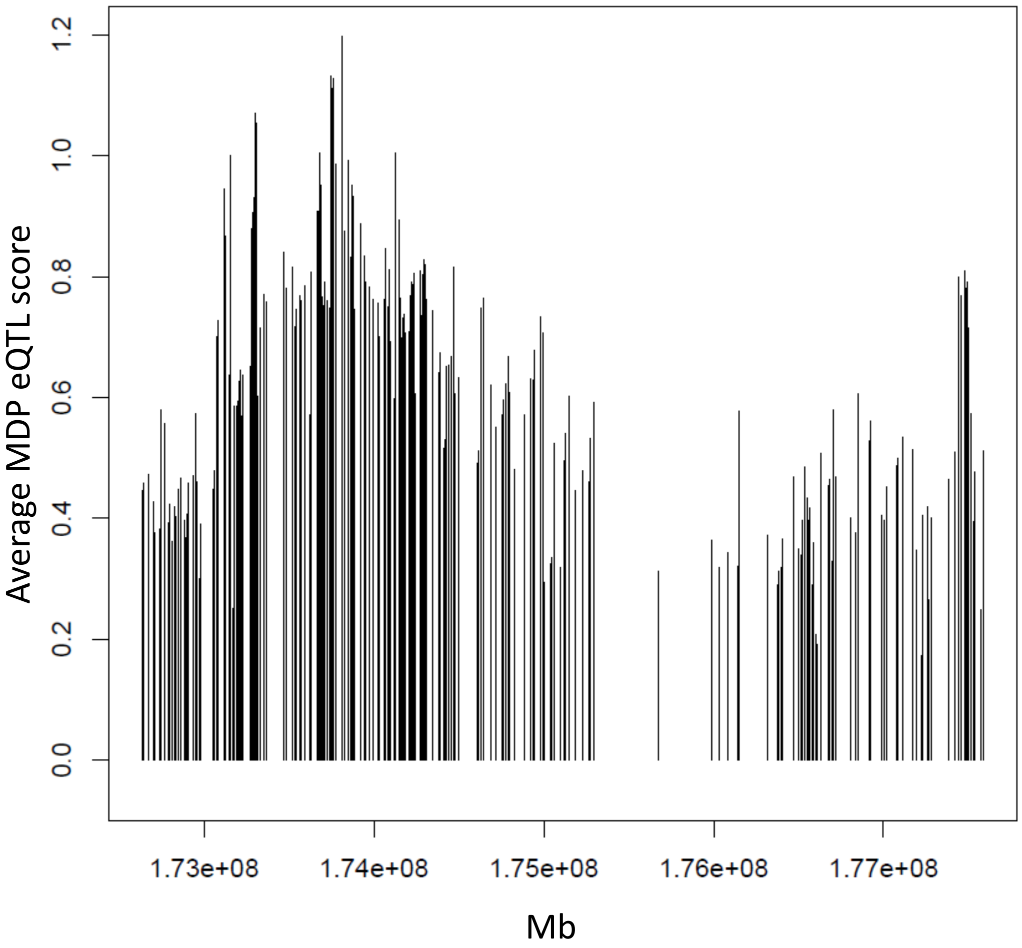

Significant in BXD: 7 genes

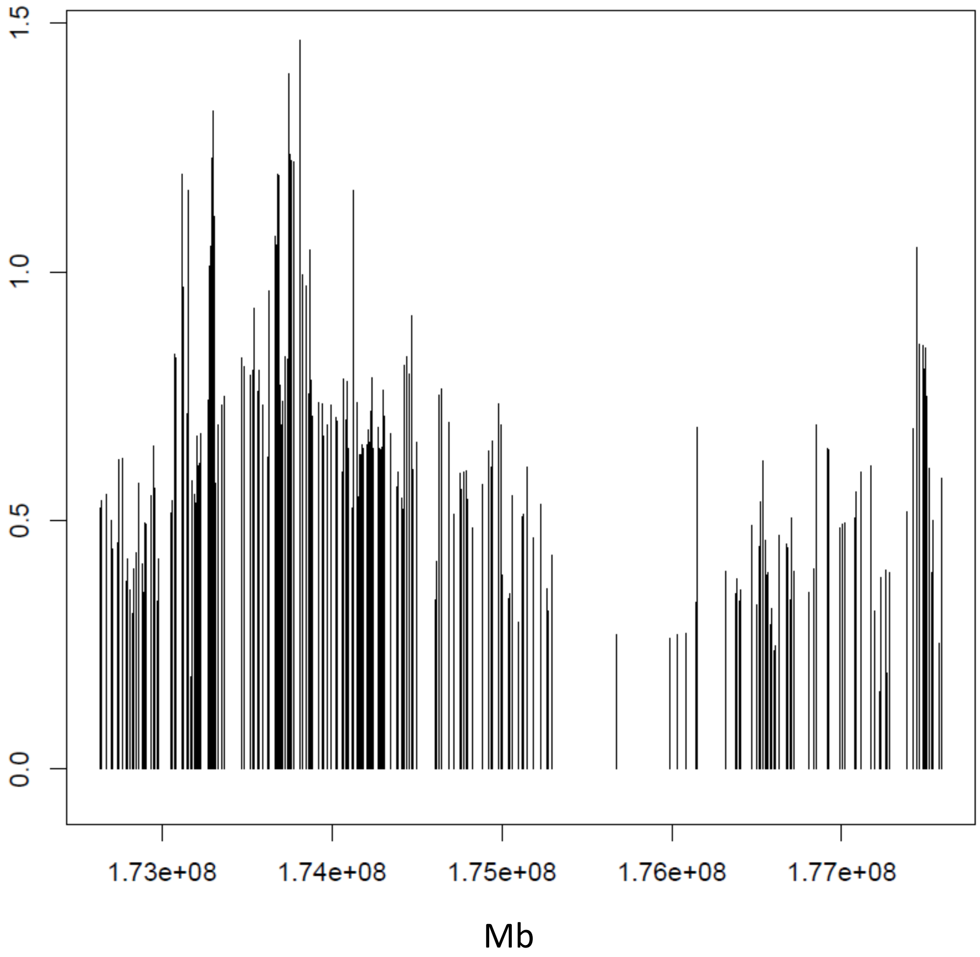

Significant in BXD and MDP: 4 genes
